# The effectiveness of various CSF diversion surgeries in idiopathic normal pressure hydrocephalus: a systematic review and meta-analysis

**DOI:** 10.1016/j.eclinm.2024.102891

**Published:** 2024-10-30

**Authors:** Ahmed Salih, Aksaan Arif, Madhur Varadpande, Rafael Tiza Fernandes, Dragan Jankovic, Darius Kalasauskas, Malte Ottenhausen, Andreas Kramer, Florian Ringel, Santhosh G. Thavarajasingam

**Affiliations:** a School of Medicine, Imperial College London, London, United Kingdom; b Imperial Brain and Spine Initiative, Imperial College London, London, United Kingdom; c Department of Neurosurgery, ULS São José, Lisbon, Portugal; d Department of Neurosurgery, University Medical Center Mainz, Mainz, Germany

**Keywords:** Idiopathic normal pressure hydrocephalus, Cerebrospinal fluid shunt, Outcomes, Meta-analysis

## Abstract

**Background:**

Idiopathic normal pressure hydrocephalus (iNPH) is commonly treated using cerebrospinal fluid (CSF) diversion procedures, most commonly ventriculoperitoneal (VP) but also lumboperitoneal (LP), ventriculoatrial (VA) shunting, and endoscopic third-ventriculostomy (ETV). Despite the prevalence of these interventions and recent advancements in iNPH diagnostic processes, there is limited up-to-date evidence regarding surgical outcomes.

**Methods:**

A systematic review and meta-analysis were conducted to analyse the effects of CSF diversion surgeries among iNPH patients. The primary outcome was efficacy of the CSF diversion procedure, defined as symptomatic improvement, and secondary outcomes included surgical complications. Several major databases were searched for original studies from inception up to June 4, 2024, which were evaluated using random-effects meta-analyses, meta-regression, and influence analyses. This study was registered with PROSPERO: CRD42023458526.

**Findings:**

Out of the 1963 studies screened, 54 were included in this review, and 4811 patients were pooled. Overall, more than 74% of patients experienced improvement after surgical treatment (95% CI: 70–78%). VP shunting demonstrated an efficacy of 75% (95% CI 70–79%), VA shunting at 75% (95% CI: 70–80%), and LP shunting at 70% (95% CI: 52–83%). ETV had a success rate of 69% (95% CI: 58–78%). Gait improvement was high at 72% (95% CI: 67–77%), while urinary and cognitive dysfunction each improved in approximately 50% of patients. The efficacy of surgery did not increase between 2005 and 2024 (p = 0.54). Complications occurred in 20.6% of cases, with a surgery revision rate of 15.1%.

**Interpretation:**

This meta-analysis found that the overall efficacy of CSF diversion procedures for iNPH remained unchanged from 2005 to 2024, with 74% of cases showing improvement. No procedure was found to be clearly superior, and only half of the patients saw improvements in urinary and cognitive dysfunction. The stagnant efficacy over time and frequent complications highlight the need for improved patient selection criteria to best identify those most likely to benefit from CSF shunting.

**Funding:**

None for this study.

**Research in context**

**Evidence before this study**

Idiopathic normal pressure hydrocephalus (iNPH) is a progressive neurological disease considered reversible by cerebrospinal fluid (CSF) shunting. Despite numerous studies on surgical outcomes, the efficacy and complication rates remain unclear with varied reporting and success dependent on several factors such as patient selection. We conducted a literature search on PubMed, Embase, and the Cochrane Database of Systematic Reviews up to June 4th, 2024, to identify meta-analyses on the efficacy of various CSF diversion procedures in iNPH management. Search terms included ‘idiopathic normal pressure hydrocephalus’, ‘surgery’, and ‘CSF shunt’. Two studies were identified, revealing a gap in reported data across CSF diversion techniques. One study focused on ventriculoperitoneal (VP) shunt procedures, while the other offered limited insight into endoscopic third-ventriculostomy (ETV) due to limited data. Reported outcomes varied significantly and data collection up was up to 2017. Since then, there have been advancements in iNPH research, including improved precision through invasive tools, updated clinical consensus diagnostic criteria, and advancements in shunt technology, necessitating an updated evaluation of CSF diversion procedures in iNPH management. Furthermore, our study provides a unique perspective on the evolving success of CSF diversion over time, reflecting dynamic evidence in the literature.

**Added value of this study**

This systematic review and meta-analysis, including data from 4811 iNPH patients, demonstrates a consistent improvement rate of 74% across various CSF diversion surgeries, with no specific procedure shown to be superior. Surgery was largely effective in improving gait disturbances with moderate incidences of complications observed. Despite these positive outcomes, the lack of improvement in success rates over the past 19 years emphasises the necessity for enhanced patient selection.

**Implications of all the available evidence**

Our study provides an important and landmark update regarding the clinical efficacy of CSF diversion procedures, with further evaluation of the change of related literature over time. All CSF shunt surgeries have been effective in reversing symptoms of iNPH. However, these procedures carry a risk of complications, some of which are severe and require careful consideration. The stagnant efficiency over time highlights the gap in current iNPH research: the challenge of accurately identifying patients who are most likely to respond from CSF shunting. Addressing this issue may necessitate the adoption of advanced diagnostic techniques.

## Introduction

Idiopathic normal pressure hydrocephalus (iNPH) is a progressive neurological syndrome characterised by gait disturbance, cognitive impairment, and urinary incontinence concomitant with dilated cerebral ventricles under normal cerebrospinal fluid (CSF) pressure.^1^ The prevalence of iNPH is estimated to be between 10 and 22 per 100,000, and this is markedly increased within specific, often vulnerable patient subgroups, such as those residing in extended-care facilities.^2^^,^^3^ CSF diversion shunts are considered the gold-standard treatment for iNPH.^4^ Several shunt options exist, including ventriculoperitoneal (VP) which is widely favoured, lumboperitoneal (LP), that sees significant usage in certain regions and, in fewer cases, ventriculoatrial (VA) shunts. Endoscopic third-ventriculostomy (ETV) is a suggested alternative proposed to work through systolic CSF outflow in the subarachnoid space, although the efficacy of this is disputed.^5^ Despite improvements in iNPH diagnostic imaging and CSF dynamics assessment, the precise magnitude of therapeutic benefit from CSF diversion procedures is yet to be fully elucidated with varied success reported.^6^, ^7^, ^8^ Moreover, shunt surgeries are associated with significant complications, including infections and intracranial haematomas, the incidences of which are inconsistent across reports.^9^, ^10^, ^11^

Diagnosing iNPH further complicates management due to the absence of a definitive test. Guideline statements, that is the 2021 Japanese guidelines and the 2005 American–European guidelines, diverge on several factors, including age limits and parameter thresholds to differentiate iNPH from other neurodegenerative conditions.^12^^,^^13^ Variability in interpreting these criteria may lead to diverse diagnostic practices and therefore shunt surgery suitability, which, in turn, may impact treatment outcomes among studies.^14^

Recent years have seen a notable expansion in evidence for CSF shunting, and to a lesser extent, ETV in iNPH patients. This growing dataset provides an opportunity to re-evaluate the effectiveness of these surgical interventions. Moreover, advancements in patient selection methodologies, enriched by clinical tests and radiological assessments, have improved the identification of candidates most likely to benefit from shunt surgery.^15^^,^^16^ The latest consensus guidelines further highlight this by advocating for a nuanced diagnostic algorithm and the use of CSF biomarkers to predict surgical outcomes, ensuring that interventions are tailored to those with the greatest potential for improvement.^12^

Considering these developments, this systematic review and meta-analysis seeks to update the existing literature and additionally evaluate the temporal trends in CSF surgery efficacy. By integrating the most current research, we aim to report the relative benefits and risks of surgical CSF diversion techniques in the management of iNPH.

## Methods

### Literature search

This systematic review and meta-analysis was conducted using the Cochrane Collaboration and Preferred Reporting Items for Systematic Reviews and Meta-Analyses (PRISMA) guidelines and is registered with the international prospective register of systematic reviews (PROSPERO identification number: CRD42023458526).^17^ A comprehensive search from inception to June 4, 2024, was conducted on MEDLINE, Scopus, Embase, Web of Science, Cochrane Library of Registered Clinical Trials, and World Health Organisation International Clinical Trials Registry Platform. The search string consisted of the terms ‘idiopathic normal pressure hydrocephalus’ and ‘surgery’, ‘shunt’ or specific CSF diversion procedure (see Supplementary Table S1 for the full search strategy). Additional articles were also identified by manual searching.

### Study inclusion and exclusion criteria

**Fig. 1:**
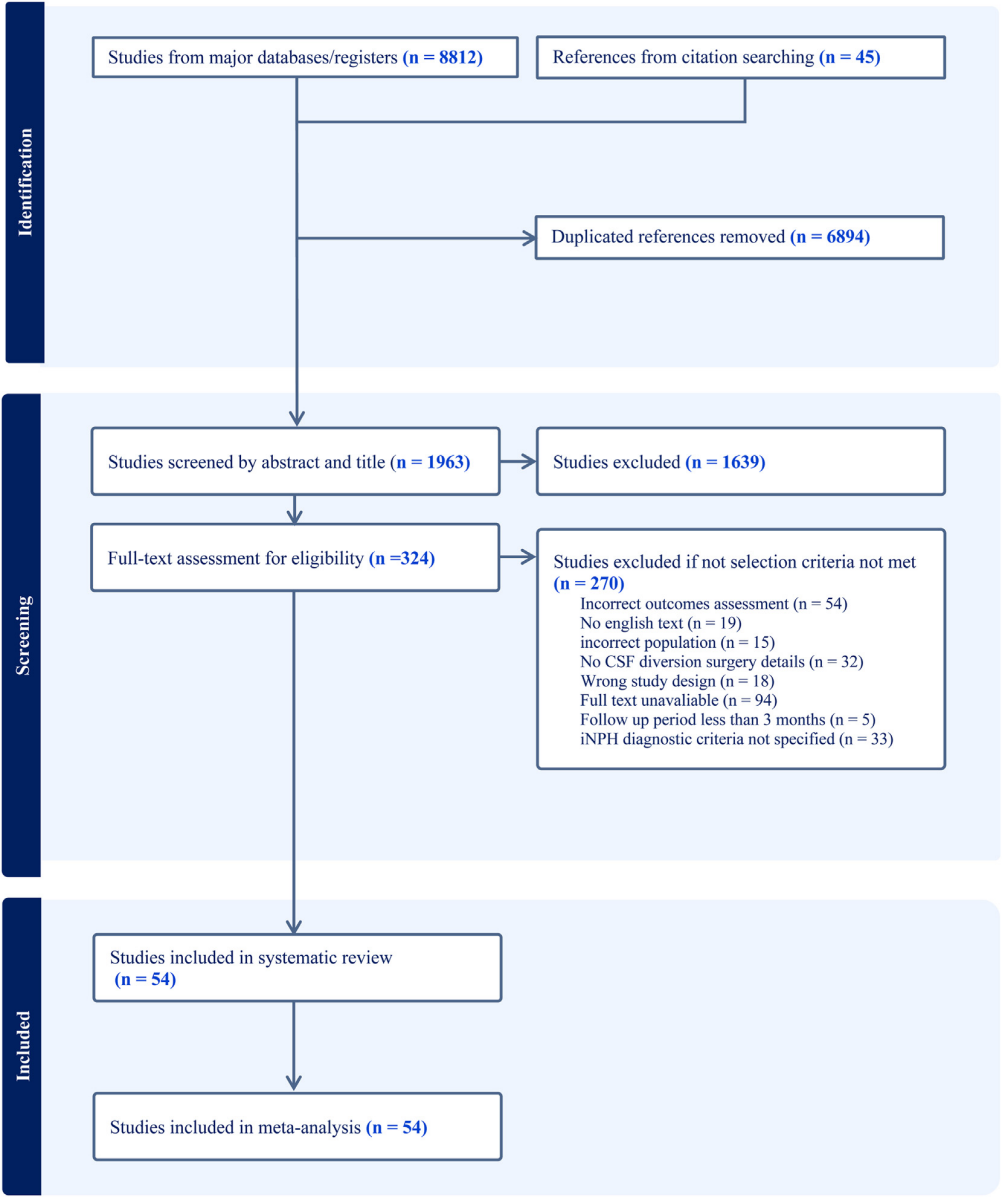
**The preferred reporting items for systematic reviews and meta-analyses (PRISMA) flowchart outlining the study selection process**.

The completed PRISMA flowchart is shown in Fig. 1. Abstracts of articles were screened for the following criteria: 1) An iNPH population or mixed hydrocephalus populations that have separate analyses of iNPH patients. 2) Use of CSF diversion surgery. Exclusion criteria include paediatric hydrocephalus, non-English articles, conference abstracts, case reports and case series (*n* < 5). Full text screening followed the strict inclusion criteria detailed below.

#### iNPH diagnostic criteria

• At least one symptom of the Hakim’s triad: gait disturbance, cognitive impairment, and urinary incontinence
• Aforementioned clinical symptoms cannot be explained by other neurological or non-neurological diseases
• Mean age of study >60 years (to incorporate the two established iNPH guidelines)^12^^,^^13^
• Radiological confirmation of ventriculomegaly (Evans index > 0.30)
• Positive results on additional invasive diagnostic iNPH tools based on either CSF hydrodynamics, intracranial pressure, or CSF evacuation (for example CSF tap test, extended lumbar drainage, infusion testing, and intracranial pressure monitoring).

#### Outcome measures

Primary—improvement on an outcome assessment scale.

• Measured at baseline and at least three months after the date of surgery in all patients
• The outcome assessment must measure either the degree of disability, neurological morbidity, or symptoms of Hakim’s triad. Supplementary Table S2 shows a comprehensive list of outcome measures utilised as a reference list during the screening.

Secondary–complications of surgery and surgical revision rates (see Supplementary Table S3).

Studies were excluded if the surgical procedure was not clearly defined, or if patients had secondary causes of normal-pressure hydrocephalus or neurological co-morbidities that could affect shunt response. Study authors were contacted to obtain missing data.

### Eligibility assessment and data extraction

References were imported into the Covidence screening tool, and duplicates were removed. Studies were evaluated for eligibility by three reviewers (AS, AA, MV).^18^ Conflicts of agreement were resolved by discussion with an independent reviewer (SGT). The relevant data was extracted using the Covidence data collection tool.^18^ Relevant data included study type, number of patients undergoing surgery, study methodology, iNPH diagnostic criteria, follow-up data, criteria for clinical improvement, outcomes assessment method, and complications. When a single study had several follow-up periods, the one with the largest sample size and, hence, minimal loss to follow-up was extracted. Additionally, when multiple reports from the same study were present, they were linked together.

### Quality assessment and data analysis

All articles were critically appraised using an adapted version of Newcastle–Ottawa Score (NOS) by three independent reviewers (AS, AA, MV), and a consensus was reached by discussion with an independent reviewer (SGT).^19^ Scores were assigned for three domains: selection criteria, comparability, and outcome with an overall score out of 9 (see Supplementary Table S4). For each study included, the risk of bias was deemed to be either high, with some concern or low.

An Egger’s regression and asymmetry test was used to assess publication bias (p < 0.05 was deemed significant).^20^ If publication bias was detected, the trim-and-fill method was used to estimate the effect size impact. A proportional meta-analysis was conducted for each CSF diversion procedure for symptomatic improvement, which was calculated as the number of improved patients divided by sample size. We also analysed surgical complications, including a separate analysis for surgery revision rates. Given the anticipated study heterogeneity, we utilised a random-effects model. The inverse variance method was used for pooling effect sizes. The Hartung-Knapp method was used to adjust test statistics and confidence intervals. Heterogeneity was estimated using the chi-squared statistic (I^2^) with the associated p-value in addition to visual confirmation from forest plots.

To assess whether key clinical and methodological factors impact the symptomatic improvement efficacy, several meta-regression analyses were conducted. The dependent variable was the proportion of surgery responders. Predictor variables considered included baseline characteristics, iNPH disease metrics, and shunt details. These predictors were based on expert opinion and data availability. Consistency was maintained by employing the same estimate method of study variance in meta-regressions as in meta-analyses.

An influence sensitivity analysis was carried out to identify studies that significantly contribute to the diversity between studies in the meta-analysis (e.g., outliers). A leave-one-out method was used to generate Baujat plots, influence diagnostics, and pooled leave-one-out analysis. In instances where outliers were identified, the meta-analysis was recalculated to exclude them.

Data preparation, statistical analysis, and forest plot synthesis were carried out using the ‘meta’ and ‘metafor’ packages on R software (version 4.3.2).

### Role of funding source

There was no funding source for this study.

## Results

**Fig. 2:**
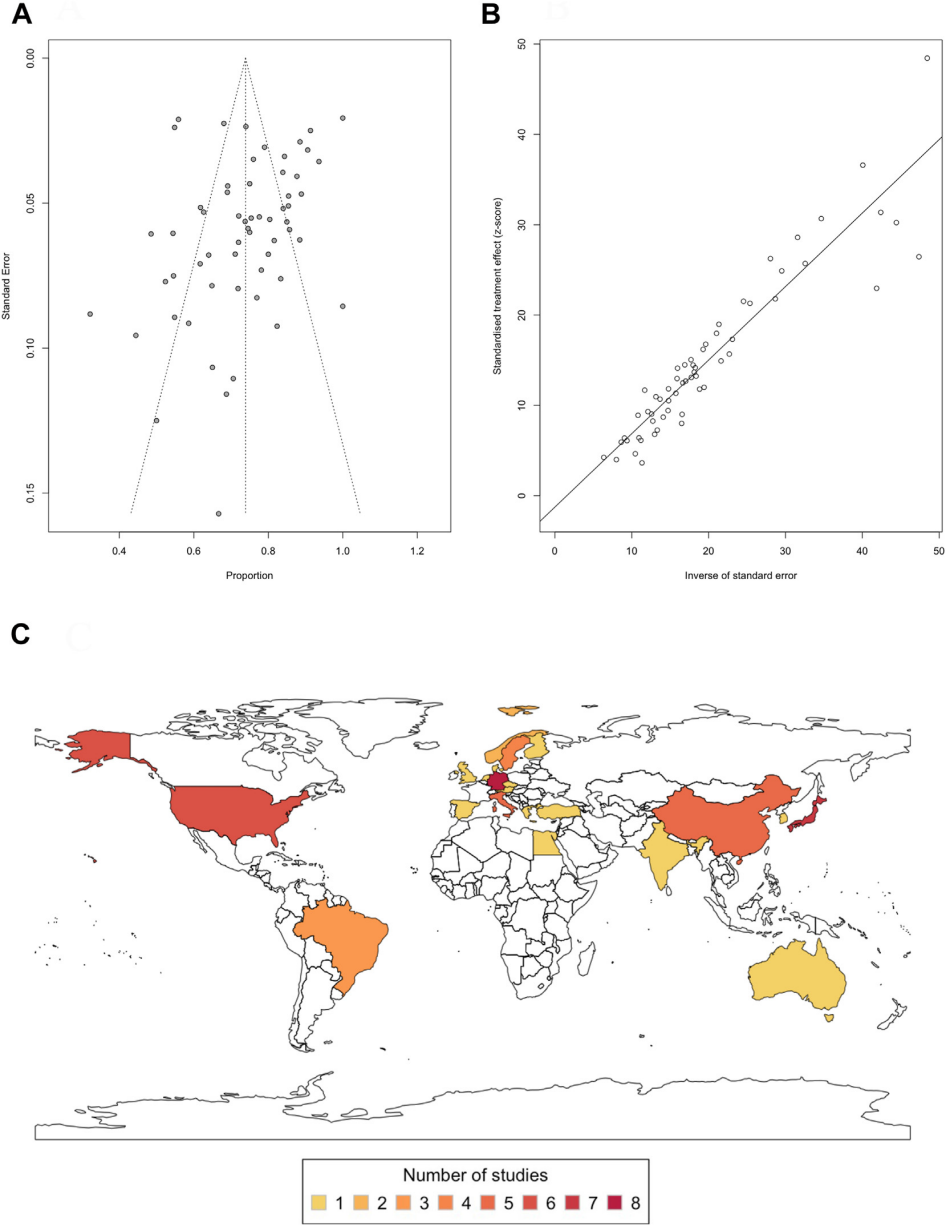
**Publication bias testing and distribution of included studies on a world map. (A)** A funnel plot is shown, which plots every study included in the meta-analysis (n = 54). Their observed effect sizes (Proportions) are on the x-axis, against a measure of their standard error on the y-axis. **(B)** An Egger’s asymmetry test funnel plot of all data points included in the meta-analysis (n = 41). p-value < 0.05 is deemed significant and implicates publication bias. Egger’s asymmetry test yielded p = 0.1761, calculated by running an Egger’s regression (see Egger’s regression line) on the collated standard errors of all data used in the meta-analysis. **(C)** A world map indicating the origin of publications of included studies (n = 53), not including Kingle et al.^43^ as this was a European multicentre study. Sæhle et al.^34^ investigation took part in 2 countries: Sweden and Norway. The countries are coloured according to the number of studies included in this systematic review. The legend at the bottom indicates the colour coding. The following countries are coloured: Germany (n = 8), Japan (n = 7), United States of America (n = 6), Italy (n = 5), China (n = 5), Sweden (n = 4), Brazil (n = 4), Norway (n = 2), and one study each from the United Kingdom, The Netherlands, Spain, Austria, Denmark, South Korea, India, Greece, Finland, Egypt, the Czech Republic, Australia, and Turkey.

Of the 1963 studies reviewed, 54 papers met the inclusion criteria and were included in this systematic review and meta-analysis^10^^,^^11^^,^^21^, ^22^, ^23^, ^24^, ^25^, ^26^, ^27^, ^28^, ^29^, ^30^, ^31^, ^32^, ^33^, ^34^, ^35^, ^36^, ^37^, ^38^, ^39^, ^40^, ^41^, ^42^, ^43^, ^44^, ^45^^,^^46^, ^47^, ^48^, ^49^, ^50^, ^51^, ^52^, ^53^, ^54^, ^55^, ^56^, ^57^, ^58^, ^59^, ^60^, ^61^, ^62^, ^63^, ^64^, ^65^, ^66^, ^67^, ^68^, ^69^, ^70^, ^71^, ^72^ (Fig. 1). The combined analysis comprised of 4811 iNPH patients. Using the modified Newcastle-Ottawa Scale, 53 studies were considered either low bias or with some concern, while one study scored as high risk^30^ (Supplementary Fig. S1). The Egger’s asymmetry plot found no significant publication bias overall (p = 0.18) (Fig. 2A and B), and this lack of bias was consistent across each CSF diversion procedure (Supplementary Fig. S2).

### Study type and design

Three (5.6%) studies were randomised parallel trials, while 19 (35.1%) were prospective and, the majority, 32 (59.3%) adopted a retrospective design. Out of the 54 studies, the most common countries of study origin were: Germany (14.8%), Japan (13.0%) and the United States of America (11.1%) (Fig. 2C). Regarding treatment modalities, 35 studies solely investigated VP shunting (n = 2442). 7 studies focused on LP shunting (n = 333), whereas 3 studies were on VA shunting (n = 152), and 5 studies investigated ETV (n = 159). In 4 studies, several CSF diversion surgeries were compared, encompassing 1725 patients. Among these, 906 cases were with a VP shunt, 636 with LP shunt, 167 with VA shunt and 16 with ETV. Out of the 49 studies using a shunt, most studies (n = 29) utilised a programmable shunt valve, whereas a fixed shunt valve was only used in 6 studies. A combination of programmable and fixed valve was used in 7 studies, and the rest did not specify (n = 7). The most frequently employed valves were Codman Hakim adjustable valve (Codman) (24.8%), Strata (Medtronic) (12.1%), and proGAV (Miethke and Aesculap) (11.9%).

### Patient characteristics

The mean age was 72 years with 40.9% of participants being female. Regarding clinical presentation, 90.2% exhibited gait abnormalities. Impaired cognition was observed in 71.2% of participants, while 73.7% presented with urinary symptoms. Overall, 64.1% presented with all three components of Hakim’s triad. The average duration of iNPH symptoms was 23.3 months. The pre- and post-operative scoring systems used include: iNPH grading scale (14 studies), Mini-Mental State Examination (14 studies), modified Rankin Scale (13 studies), Japanese iNPH grading scale (11 studies), Kiefer scale score (6 studies), Black grading scale (4 studies), timed up and go test (4), quality of life assessment (4 studies) and the Tinetti test score (3 studies) (Table 1).

**Table 1:** A list of all included studies in this systematic review.

Study	Country	Study design	CSF diversion surgery	Number of participants	INPH diagnostic criteria	Scales (s) used	Criteria for surgery response	Follow up	Mean age (SD)
Agerskov et al. 2018^68^	Sweden	Retrospective single-centre	VPS	429	1) Gait or balance disturbance, cognitive disturbance or impaired urinary continence; 2) Enlarged ventricles with Evans’ index > 0.3 with supporting features; 3) Lumbar CSF opening pressure <18 mmHg; 4) Insidious onset of symptoms over at least 3 months after 40 years of age; 5) Lack of cause and no signs of CSF obstruction on MRI or radionuclide cisternography	Gait abnormalities measures, Romberg test, MMSE. Urinary incontinence graded 1–6	Composite score: ≥5 points postoperatively	3–6 months	71 (9.5)
Aruga et al. 2018^57^	Japan	Retrospective single-centre	LPS	48	1) Age > 60 years; 2) Presence of ≥1 symptom of the clinical triad: gait disturbance, cognitive impairment, and urinary incontinence; 3) Presence of ventricular dilatation: Evans’s index > 0.3 on CT or MRI; 4) Clinical triad symptoms not explained by other neurological diseases; 5) Normal CSF pressure	Overactive Bladder Symptoms Score, Quality of Life index, Urodynamic studies (filling cystometry and pressure-flow studies)	Symptomatic change was graded on a five-level scale and the highest two grades were regarded as improvement.	average 3 months	79
Bech-Azeddine et al. 2007^67^	Denmark	Prospective single-centre	VPS	28	1) Presence of >2 symptoms from the clinical triad; 2) Ventricular enlargement on a cerebral CT scan; 3) Increased Rout (>10 mm Hg/ml/min) with or without a B-wave activity ≥ 50% of the monitoring period.	MMSE, Hachinski Ischaemic Score, Global Deterioration	An increase of at least two degrees in the ordinal scales	3–9 months	64
Belotti et al. 2022^66^	Italy	Retrospective single centre	VPS	45	Based on American-European iNPH guidelines	Short Form 12 Health Survey, EQ-5D questionnaire.	Improvement in Quality of Life	12 months	
Bloch et al. 2012^56^	United States	Retrospective single-centre	LPS	33	1) Patients with ≥1 of the 3 cardinal symptoms of iNPH (gait instability, incontinence, or dementia); 2) Enlarged ventricles on preoperative intracranial imaging; 3) Positive improvement in symptoms with lumbar puncture or extended lumbar drainage.	30-foot gait assessment. Evaluation of gait quality	Improvement from baseline	average 19 months	72.6
Chen et al. 2022^55^	China	Prospective single-centre	VA	47	1) Age ≥ 60 years; 2) ≥ 1 clinical manifestations of the triad of gait disorder, cognitive impairment, and urinary incontinence; 3) Manifestations of ventricular enlargement (EI ≥ 0.3) on imaging; 4) CSF pressure ≤ 200 mmH2O on lumbar puncture, with normal CSF biochemical results; 5) Positive response on the CSF tap test	mRS, iNPHGS	mRS: ≥ 1 points, total iNPH score: ≥ 1 point	12 months	69.2 (5.9)
Delwel et al. 2013^65^	The Netherlands	Multicentre randomised trial	VPS	52	1) 2 or more symptoms of the NPH triad (gait disturbance, cognitive dysfunction and urinary dysfunction); 2) Hydrocephalus on CT scans; 3) CSF pressure <15 cm H2O	Boon gait score, 3MS	Boon gait score and/or 3MS: ≥15%	9 months	
Eshra 2013.^53^	Egypt	Prospective single-centre	ETV	16	1) Age ≥ 50 years old; 2) Patients with 2 ≥ symptoms of the classic triad of dementia, gait disturbance and urinary incontinence; 3) All patients had ventricular dilatation on brain imaging; 4) Negative history of infection, brain injury or haemorrhage; 5) ICP were less than 15 cm H2O (8–14 cm H2O); 6) Tap test positive response	JNPHGS	≥1 point	7–26 months	
Fang et al. 2022^52^	China	Retrospective single-centre	LPS	85	1) Age ≥ 60 years; 2) Presence of gait/balance disorder and ≥1 other symptoms of the triad; 3) CT or MRI showing ventriculomegaly (Evans’ index of >0.3); 4) Absence of known disorders causing ventriculomegaly; 5) Normal CSF opening pressure (≤20 cm H2O); 6) Positive response to the CSF Tap Test	mRS, iNPHGS, MMSE, TUG	mRS: 1 point, INPHGS (gait/cognition/urinary): 1 point, TUG 3M: 10%, MMSE: >2 point	12 months	74.7 (7.1)
Foss et al., 2007.^64^	Norway	Retrospective single-centre	VPS	27	1) Clinical evaluation (minimum two of: gait disturbance, cognitive dysfunction or urinary incontinence); 2) Radiological evaluation with Evan’s index ≥ 0.3; 3) ICPM measurments with an ICP mean of <15 mm g.	MMSE, DRS	4-point improvement in MMSE, or improvement by 1 SD in 50% of the DRS subtests	6–9 months	72
Fountas et al. 2012^21^	Greece	Prospective single-centre	ETV	7	1) Age < 80 years; 2) Duration of symptoms <6 months; 3) Absence of any other clinically evident comorbidity; 4) Hyperdynamic CSF flow in the aqueduct demonstrated on the preoperative cine MRI study (aqueductal CSF stroke volume >42 m L); 5) symptom improvement after umbar CSF drain test, and 7) no previous shunt insertions.	MMSE, JNPHGS	Postoperative improvement in clinical grade	12 months	72.2 (3.14)
Freimann et al. 2013^63^	Germany	Retrospective single-centre	VPS	100	1) Gait disturbance, mental deterioration and/or urinary incontinence as symptoms; 2) Cranial imaging found enlarged ventricles (Evan’s index > 0.3), effaced cortical sulci and missing signs of severe cortical atrophy; 3) Patient improvement of at least gait disturbance following CSF drainage	Black	fair to excellent improvement	12 months	72
Gala et al. 2017^62^	Spain	Retrospective single-centre	VPS	29	1) Age ≥ 60; 2) Presence of ≥2 symptoms from the classic adult chronic idiopathic hydrocephalus triad; 3) Evans index > 0.3; 4) Normal CSF and CSF pressure < 20 mm H2O; 5) Clinical improvements after lumbar puncture or CSF drainage.	Klassen	At least partial improvement	12 months	72.9 (7.9)
Gangemi et al. 2008^58^	Italy	Retrospective multicentre	ETV	110	1) At least two symptoms of the Adams-Hakim triad; 2) Clinical history negative for secondary causes 3) Ventricular enlargement on MRI; 4) ICP between 8 and 12 mmHg.	JNPHGS	≥1 point	2 years	67
Gölz et al. 2014^61^	Germany	Retrospective single-centre	VPS	61	1) Patients with possible symptoms of iNPH (gait disturbance, cognitive dysfunction and urinary dysfunction); 2) Ventriculomegaly on CT or MRI (Evans Index ≥ 0.3). 3) Elevated resistance (Rout > 13 mm Hg/[ml/min]) on intrathecal infusion test; 4) Improvement of at least 20% in walking speed and step count after CSF tap test.	Kiefer score	NPH recovery rate ≥2 points	6 years	64
Grasso et al. 2019^60^	Italy	Retrospective single-centre	VPS	50	1) Patients with ventriculomegaly (Evans index ≥ 0.3, assessed on MRI/CT) 2) Patients have ≥ 1 of 3 cardinal clinical features of iNPH. 3) Positive response spinal tap testing (defined as an objective improvement in gait, cognition or bladder control)	JNPHGS	≥1 point	up to 10 years	71 (8.5)
Grasso et al. 2023^59^	Italy	Retrospective single-centre	VPS	127	Patients with ventriculomegaly (Evans index ≥ 0.3, assessed on MRI/CT) and who have ≥ 1 of 3 cardinal clinical features of iNPH were considered for a spinal tap test. A positive spinal tap test was suggestive of iNPH.	SF-35, iNPHGS, 10MWT, mRS	1 point in total iNPH score, >0.1 m/s in gait speed	average 118.5 months	69 (5.7)
Hailong et al. 2008^25^	China	Retrospective single-centre	ETV	17	1) Presence of ≥2/3 classic symptoms of the Hakim triad 2) Evidence of communicating hydrocephalus with enlarged ventricles on neuroimaging; 3) Isotope clearance impairment and ventricular retrograde flow on radionuclide cisternography; 4) Signs of high velocity flow on midbrain aqueduct on T2-weighted MRI; and 5) CSF pressure assessment and infusion/tap test.	Kiefer score	(Preoperative − postoperative Kiefer score)/preoperative Kiefer score: >5 RR	average 14 months	65.9 (10.1)
Hong et al. 2018^51^	South Korea	Retrospective multicentre	VPS	31	In accordance with the iNPH Guidelines for probable iNPH	mRS, iNPHGS	≥3 in INPH total score or ≥2 in mRS	12 months	
Hung et al. 2017^11^	United States	Retrospective single-centre	VA and VP	346 (VPS), 150 (VA)	1) Clinical criteria included presence of at least gait imbalance, or 1/3 cardinal symptoms of iNPH; 2) No ventricular obstruction on CT or MRI, and an Evans Index >0.3. 3) Diagnostic lumbar puncture with opening pressure < 25 cm of H2O in all cases	Tinetti score	Improvement on Tinetti gait score	average 15 months	74–VA. 73–VPS
Junkkari et al. 2019^50^	Finland	Prospective single-centre	VPS	175	Patients with ≥1 symptom possibly related to NPH (impaired gait, cognition or urinary continence) together with enlarged brain ventricles (Evans’ index > 0.3) in CT or MRI and without other explicit cause of the symptoms.	Walking speed, iNPHGS, HRQOL	Walking speed: >20% improvement, INPH: 1 grade, HRQOL: 0.015 improvement	3 months	
Klinge et al. 2012^49^	Europe	Prospective multicentre	VPS	115	Patients with clinical evaluation (symptoms and signs) and MRI findings compatible with iNPH underwent a CSF tap test. Resistance to CSF outflow measurements using constant rate infusion	mRS, iNPH	1 point in mRS or 5 points in total iNPH score	12 months	70
Krahulik et al. 2020^48^	Czech Republic	Prospective single-centre	VPS	61	Patients with symptoms of Hakim’s triad (gait apraxia, urinary incontinence and dementia underwent CT or MRI to identify enlarged ventricles (Evans index with >0.3) and rule out other causes of hydrocephalus.Lumbar infusion test with continuous infusion.	Incontinence frequency, MMSE	Decreased incontinence frequency, MMSE: 3 point	6 months	74.9 (5.3)
Kumar et al. 2021^69^	India	Retrospective single-centre	ETV	9	1) Clinical: progressive clinical picture of triad; 2) None of the patients had a personal history of SAH, head injury, cranial neurosurgery for any reason, or central nervous system infection; 3) Radiological: ventricular dilation, confirmed by brain CT and MRI, communicating hydrocephalus and an Evans index (EI) of at least 0.30; 4) CSF pressure within the normal range, as demonstrated by the opening pressure on lumbar CSF puncture.	JNPHGS	≥1 point	12 months	65.6 (2.8)
Kuriyama et al. 2017^47^	Japan	Retrospective multicentre	VPS, LPS and VA	434 (VPS), 553 (LPS), 17 (VA)	Based on the Guidelines for Management of Idiopathic Normal Pressure Hydrocephalus: Second Edition, 2012	mRS	mRS: obvious positive improvement	Variable	76.4 (7)
Lemcke et al. 2010^46^	Germany	Prospective single-centre	VPS	35	Patients with gait ataxia alongside other iNPH cardinal symptoms and neuroradiological evidence of ventricular enlargement underwent a dynamic intrathecal infusion test via a lumbar puncture (>13 mmHg/ml/min threshold)	Black, Kiefer scale	At least fair (partial improvement) on the Black scale	2 years	68 (11.7)
Liu et al. 2016^44^	United States	Retrospective single-centre	VA	58	1) Age ≥ 21 years; 2) Clinical symptoms suggestive of hydrocephalus; 3) No previous treatment, and an Evans Index > 0.3) MRI findings of non-obstructive hydrocephalus with normal morphology of the third ventricle; 4) Lumbar puncture prior to treatment with opening pressure < 25 cm H2O.	iNPHGS, TUG, Tinetti score, MMSE	Improvement in at least one of the symptoms of hakim’s triad	mean 16 months	74
Lundkvist et al. 2010^43^	Sweden	Retrospective single-centre	VPS	68	1) Age >40 years; 2) Insidious onset and progression over time; 3) Gait⁄balance disturbance accompanied by cognitive and⁄or urinary disturbance; 4) ICP 0.7–2.4 kPa; 5) Cranial CT or MRI findings of communicating hydrocephalus with Evan’s index >0.3) Idiopathic aetiology.	Gait speed	Gait speed increase by ≥10%	average 6.4 months	71.6 (6.4)
Meier et al. 2005^42^	Germany	Prospective multicentre	VPS	122	Patients with at least gait ataxia and extended ventricles detected by neuroradiologic imaging, underwent an intrathecal infusion test. A resistance of >13 mm Hgmin/mL was defined as pathologic. Following this, a diagnostic CSF drainage of at least 60 mL CSF was carried out.	Black, Kiefer score	NPH recovery rate ≥ 2 points	12 months	67
Miyajima et al. 2016^35^	Japan	Prospective multicentre	LPS and VPS	100 (VPS), 83 (LPS)	1) Patient age 60–85 years; 2) Triad of symptoms (gait disturbance, cognitive dysfunction, urinary incontinence) 3) Radiological evaluation: ventriculomegaly (Evans’ index > 0.3) and high-convexity and medial subarachnoid space tightness on coronal MR images; 5) Normal CSF content and pressure	mRS, iNPHGS	≥1 point	12 months	76.4 (4.7)–LPS, 74.5 (5.1)–VPS
Moriya et al. 2015^24^	Japan	Retrospective single-centre	LPS	32	Based on Japanese guidelines for iNPH	mRS, MMSE, FAB, and TMT-A	mRS: ≥ 1 point	12 months	73.7 (6.8)
Nakajima et al. 2015^38^	Japan	Retrospective single-centre	LPS	51	1) Clinical neurologic manifestations; 2) MRI findings of ventricular enlargement; 3) Tap tests evaluation criteria consistent with Japanese guidelines for iNPH.	JNPHGS, mRS	JNPHGS, mRS: 1 point improvement	12 months	75 (6.4)
Nakajima et al. 2018^40^	Japan	Retrospective single-centre	LPS	68	Based on Japanese guidelines for iNPH	mRS	≥1 point	12 months	75
Oliveira et al. 2020^54^	Brazil	Prospective single-centre	VPS	50	1) Clinical syndrome consistent with Adams-Hakim criteria (gait apraxia, urinary incontinence and dementia); 2) Ventricular dilation documented by CT and MRI 3) Clinical response to Tap Test.	JNPHGS	≥1 point	12 months	77.1 (10.9)
Oliveira et al. 2013^10^	Brazil	Prospective single-centre	VPS	24	1) One symptom of Adams-Hakim criteria (gait apraxia, urinary incontinence and dementia); 2) Ventricular dilatation documented by cranial CT and MRI scans; 3) Clinical response to Tap Test.	JNPHGS, MMSE, TUG	JSINGH: 1 point, MMSE: 3 point, TUG: 10%	12 months	77.1 (6)
Petersen et al. 2014^41^	Sweden	Prospective multicentre	VPS	37	Clinical evaluation (gait and balance disturbance and/or mental deterioration and/or bladder disturbance), MRI evaluation: enlarged ventricles (Evans index > 0.3 and evidence of an open aqueduct). A lumbar puncture was performed ICP was determined < 18 mm Hg.	iNPHGS. EQ-5D	>5 points in total iNPH score	6 months	70
Pfisterer et al. 2009^22^	Austria	Retrospective single-centre	VA	47	1) Clinical evaluation of gait disturbance, cognitive dysfunction or urinary dysfunction; 2) Enlarged ventricles on CT or MRI (Evan’s ratio >0.3) and/or periventricular lesions and/or mild flattened cortical sulci; 3) ICPM > 50 mmHg	Gait, urinary, cognition, ordinal scale	1 grade	average 6.5 years	
Pinto et al. 2013^45^	Brazil	Randomised parallel open-label trial	ETV and VPS	ETV (16), VPS (26)	1) Progressive clinical picture ≥1 of gait apraxia, cognitive impairment, and sphincter incontinence; 2) No personal history of subarachnoid hemorrhage, head trauma, cranial neurosurgery or CNS infection; 3) CT/MRI showing communicating hydrocephalus and ventricular dilation (Evans index >0.3); 4) Opening pressure on lumbar CSF puncture between 7 and 24 cm H2O.	JNPHGS	2 points	12 months	71
Popal et al. 2021^37^	China	Retrospective single-centre	VPS	68	1) Clinical: ≥ 1 aspect of the classic iNPH triad with gradual onset in adult patients; 2) Radiological: Patients with ventriculomegaly (Evans index >0.3 on cranial CT or MRI); 3) Spinal tap test (spinal-TT) positive	Krauss improvement index, mRS	>0.5 Krauss, mRS: 1 point	6 months	71.1 (8.4)
Pujari et al. 2008^23^	United States	Retrospective single-centre	VPS	55	Patients with ventriculomegaly on CT or MRI and ≥2 clinical features of NPH underwent 2 days of continuous CSF pressure monitoring in hospital, followed by a 3-day trial of controlled CSF drainage.	MMSE, Urinary frequency, Tinetti test	MMSE: 3 point, Decreased incontinence frequency, gait evaluation improvement	6 months	71.7 (9)
Sæhle et al. 2014^36^	Sweden and Norway	Randomised blinded trial	VPS	55	In accordance with the iNPH Guidelines for probable iNPH: 1) Clinical symptoms suggestive of iNPH (gait and/or balance impairments and disturbances in cognition or control in urination); 2) Radiological signs of ventriculomegaly (Evans index > 0.3); 3) Normal ICP	iNPHGS, NPH scale	iNPHGS: 5 point, NPH scale: 1 point	6 months	71
Shaw et al. 2016^39^	Australia	Prospective single-centre	VPS	40	1) Gait disturbance and at least one other area of impairment; 2) Neuroimaging demonstrated ventriculomegaly, (Evan’s index >0.3) 3) Patients underwent constant-rate CSF infusion studies or trial CSF removal via lumbar puncture tap tests	Gait time, UPDRS-III, MMSE, ACE-R	Gait time: reduction ⩾20%, MMSE: 2 points, ACE-R: ⩾5 points. UPDRS-III: reduction ⩾10	12 months	77.1 (7.2)
Shinoda et al. 2017^35^	Japan	Prospective single-centre	VPS	50	1) Age ≥ 60 years 2) Presence of ≥1 symptoms of the triad (gait disturbance, cognitive impairment, and urinary symptoms) 3) Ventriculomegaly on MRI (Evans’ index > 0.3) 4) The absence of known disorders causing ventriculomegaly; 5) Normal CSF content and pressure 6) Positive response to CSF tap test.	mRS, MMSE, TMT-A, TUG-T	mRS scale: 1 point, MMSE: 3 point, TMTA: 30%, TUG-T: 10%	12 months	77.6 (5.9)
Suchorska et al. 2015^34^	Germany	Retrospective single-centre	VPS	89	1) Clinical presentation: gait that is small-stepped or broad-based combined with cognitive impairment and/or urge incontinence; 2) Radiological: hydrocephalus as defined by Evans’ index >0.3 on CT or MRI; 3) Improvement of symptoms after CSF drainage (single puncture and/or 3 day lumbar drainage).	Black, Kiefer score	At least ‘good’ for black scale, Kiefer scale: 2 point	average 28 months	73.5 (6.3)
Sun et al. 2022^32^	China	Retrospective single-centre	VPS	65	Based on International and Japanese Guidelines	mRS, INPHGS	mRS: 1 point, INPHGS (gait/congition/urinary): 1 point	average 48 months	61.8 (11.8)
Thomas et al. 2005^31^	United States	Retrospective single-centre	VPS	42	1) Two or more clinical symptoms of NPH (gait disturbance or urinary incontinence, with cognitive impairment); 2) CT or MRI findings of hydrocephalus; 3) normal opening CSF pressure on lumbar puncture (<15 mm Hg); 4) presence of A- or B-waves on continuous CSF pressure monitoring via spinal catheter 5) improvement CSF drainage	MMSE	MMSE: 4 point	3 months	73 (10)
Thompson et al. 2017^30^	UK	Retrospective single-centre	VPS	35	1) Symptoms suggestive of iNPH (gait disturbance, dementia and urinary incontinence); 2) Enlarged ventricles on MRI (Evans index >0.3). 3) Findings from the lumbar infusion test suggestive of iNPH	10 MWT	10% improvement	average 27.5 months	84 (3.22)
Todisco et al. 2020^29^	Italy	Prospective single-centre	LPS	44	In accordance with the iNPH Guidelines for probable iNPH: 1) Clinical symptoms suggestive of iNPH; 2) radiological signs (Evans index > 0.3) of ventriculomegaly; 3) a normal ICP.	10MWT, iNPHGS	10% improvement compared to baseline in 10 m walk time	12 months	75 (5.9)
Wetzel et al. 2018^27^	Germany	Prospective single-centre	VPS	32	1) Two symptoms of the Hakim’s triad (gradual disturbance of gait, cognitive impairment and urinary incontinence); 2) Onset of symptoms in age ≥ 40; 3) Radiological signs, MRI compatible with iNPH (symmetrical ventriculomegaly, subarachnoid space tightness and Evan’s index > 0.3); 4) Placement of lumbar drain with positive symptomatic improvement	iNPHGS, MMSE	Total iNPH score: 5 points, MMSE–improvement	3 months	71.2 (7.7)
Wetzel et al. 2020^28^	Germany	Retrospective single-centre	VPS	87	1) Patients with ≥2 cardinal iNPH symptoms; 2) Compatible radiological signs such as symmetrical ventriculomegaly and subarachnoid space tightness with an Evans’ index >0.3; 3) Positive response to a 3-day extended lumbar drainage	Kiefer score	NPH recovery rate ≥ 2 points	6 months	72 (7.6)
Yerneni et al. 2021^24^	United States	Retrospective single-centre	LPS	20	1) Patients with ≥1 of the 3 classic symptoms of iNPH (incontinence, gait instability, or dementia); 2) Intracranial imaging found enlarged ventricles; 3) A three-day lumbar drain trial finding positive improvement in symptoms.	Berg scale	1 point improvement	average 15 months	72.6 (5.42)
Reis et al. 2023^70^	Brazil	Prospective single-centre	VPS	38	In accordance with the iNPH Guidelines for probable iNPH: 1) Clinical symptoms suggestive of iNPH; 2) radiological signs (Evans index > 0.3) of ventriculomegaly; 3) a normal ICP	JNPHGS, MMSE and TUG	Improvement in JNPHGS	12 months	75.8
Goertz et al. 2024^71^	Germany	Prospective single-centre	VPS	45	(1) Presence of at least two symptoms from the Hakim triad (2) Onset of symptoms after the age of 40, with gradual worsening over a period of at least 3 months. (3) Radiological evidence, obtained MRI or CT consistent with iNPH, characterized by symmetrical ventriculomegaly and narrowing of the subarachnoid space with an Evan’s index greater than 0.3.	iNPHGS	≥5 points	12 months	
Türkkan et al. 2023^72^	Turkey	Retrospective single-centre	VPS	26	Patients with ventriculomegaly (Evan’s index > 3) were further examined. The classical clinical triad were assessed by 10MWT, MMSE and self- or carer-reported symptoms, respectively. A supportive lumbar tap test was used to guide surgical decisions based on subjective improvement.	10MWT, MMSE	10MWT: ≥20% improvement, MMSE: ≥2 points	6 months	60.3 (15.4)

An overview is provided on study design, iNPH diagnostic criteria, surgery response specification, and follow-up all included studies.

VPS: Ventriculoperitoneal shunt. LPS: Lumboperitoneal shunt. VA: Ventriculoatrial. ETV: Endoscopic Third-Ventriculostomy. mRS: modified Ranking Scale. OABSS: Overactive Bladder Symptoms Score. iNPHGS: idiopathic normal pressure hydrocephalus grading scale. MMSE: Mini-Mental State Examination. TUG: Timed Up & Go. SF-12: Short Form 12 Health Survey. QOL: Quality of Life. 3MS: modified Mini Mental Test. JNPHGS: Japanese Society of Idiopathic Normal Pressure Hydrocephalus. 10MWT: 10 Metre Walk Test. DRS: Dementia Rating Scale. ACE-R: Addenbrooke’s Cognitive Examination-Revised (ACE-R). UPDRS: unified Parkinson’s disease rating scale. TT: Tap Test. MRI: Magnetic Resonance Imaging. CT: Computed Tomography. EI: Evans’ Index. SAH: subarachnoid haemorrhage. UPDRS: unified Parkinson’s disease rating scale.

### Meta-analysis

#### Symptom improvement

**Fig. 3:**
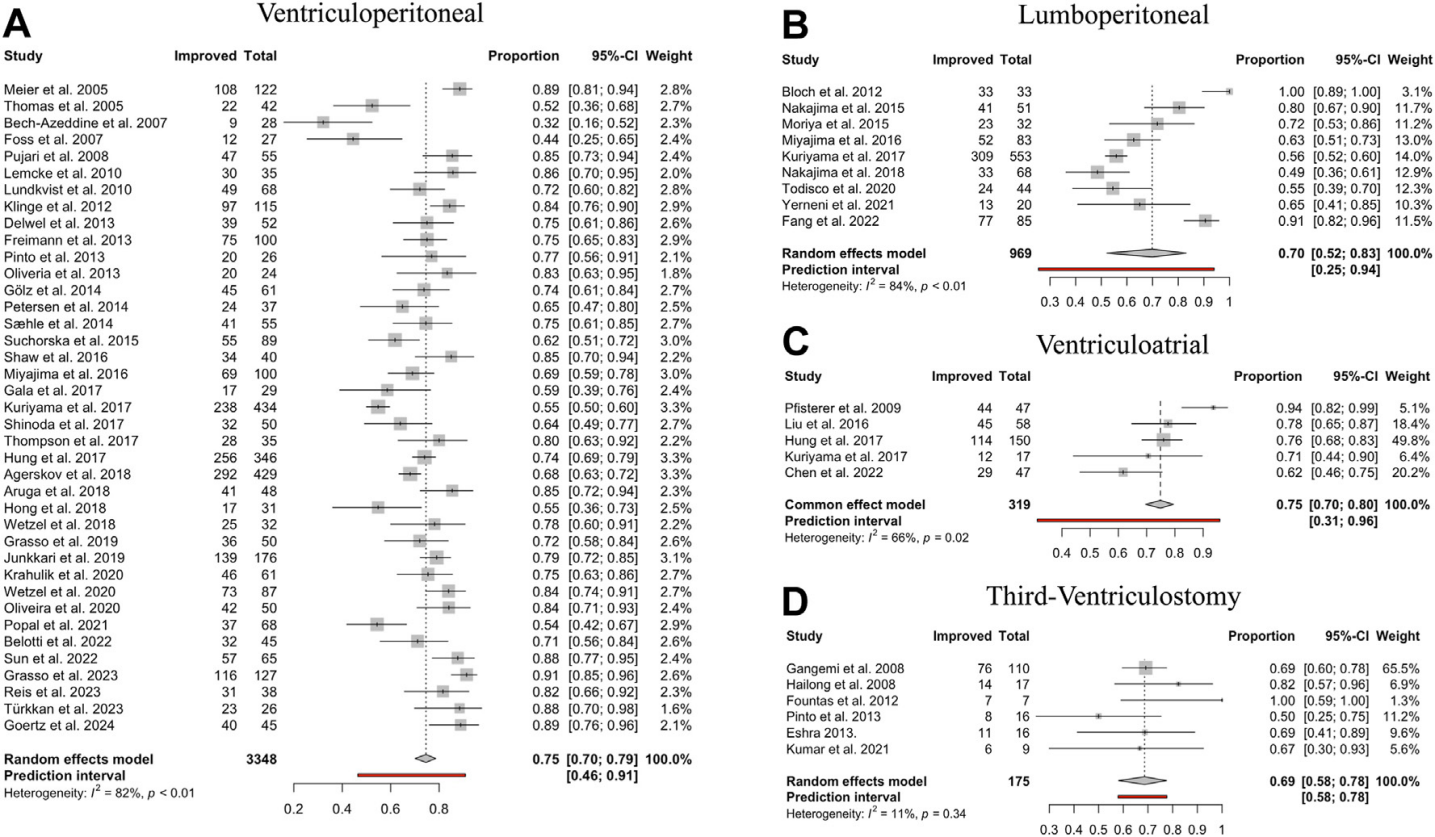
**Symptomatic improvement by CSF diversion method. (A–D)** Forest plots visualising the pooled proportional improvement of iNPH patients following CSF diversion for each surgical procedure in the order of: “Ventriculoperitoneal”, “Lumboperitoneal”, “Ventriculoatrial” and “Third-Ventriculostomy.” The size of the grey square of the “Proportions” visual correlates to study sample size and the horizontal lines indicates the confidence interval. The diamond at the bottom indicates the overall pooled proportion. The red bar below indicates the prediction interval. Heterogeneity is indicated by the chi-squared statistic (I^2^) with associated r^2^ and p-value. The 95% confidence intervals (CI) are shown in squared brackets ([ ]). The weighting of each study is by percentage (%). p-value < 0.05 is deemed significant. Furthermore, for every study, the following are displayed: study author with publication date (“Study”), total sample size number for each study (“Total”), and number of clinically improved patients (“Improved”).

For all CSF diversion surgeries, a favourable outcome (defined as an improvement on a scoring scale measured before and after surgery) was observed in 74% of patients (95% CI: 70–78%). Stratification by treatment type found positive outcomes in 75% of patients (95% CI: 70–78%) after VP shunting, 75% (95% CI: 70–80%) after VA shunting, 70% (95% CI: 52–83%) after LP shunting, and 69% (95% CI: 58–78%) after ETV (Fig. 3). The overlap between confidence intervals indicates a lack of difference between the pooled estimates.

**Fig. 4:**
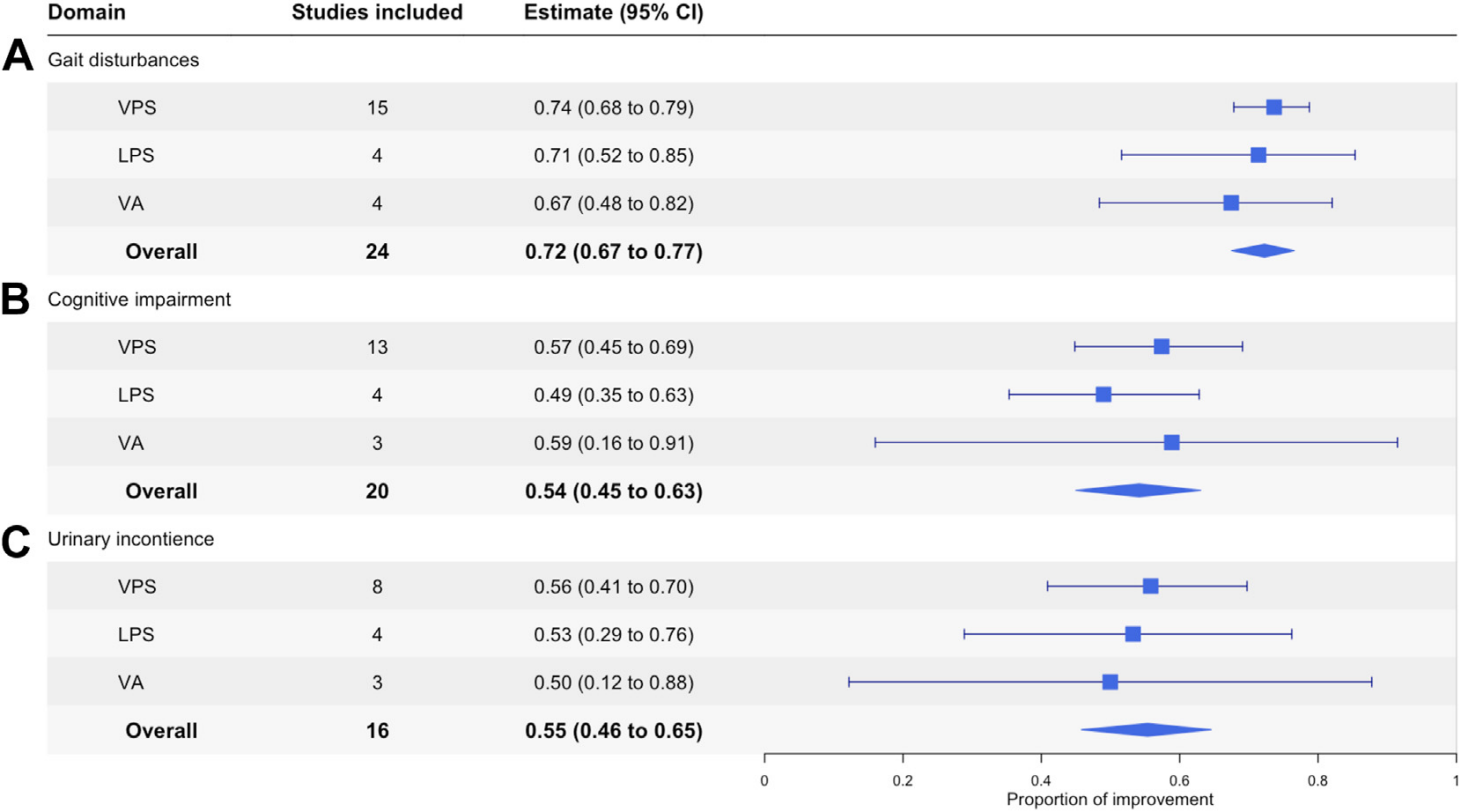
**CSF diversion procedure improvement by Hakim’s triad of symptoms. (A–C)** Forest plots visualising the proportional improvement of iNPH patients stratified by symptoms of Hakim’s triad in the order of: “Gait”, “Urinary” and “Congition” following each shunt CSF diversion (VPS, LPS and VA) and overall. The box represents the estimate with the 95% confidence interval shown as whiskers. The diamonds indicates the overall pooled proportion. The pooled estimate was obtained by proportional random effect meta-analysis where applicable.

When outcomes were categorised based on symptoms of Hakim’s triad (gait, cognition, and urinary symptoms), gait showed a high proportional improvement, occurring in 72% of patients (95% CI: 67–77%), followed by improvements in cognition in 55% of patients (95% CI: 46–65%) and urinary symptoms in 54% of patients (95% CI: 45–63%) (Fig. 4). Across the shunt types —VP, LP, or VA— the improvement for gait ranges from 67 to 74%, cognition from 49 to 59% and urinary symptoms from 50 to 56% (Fig. 4).

**Fig. 5:**
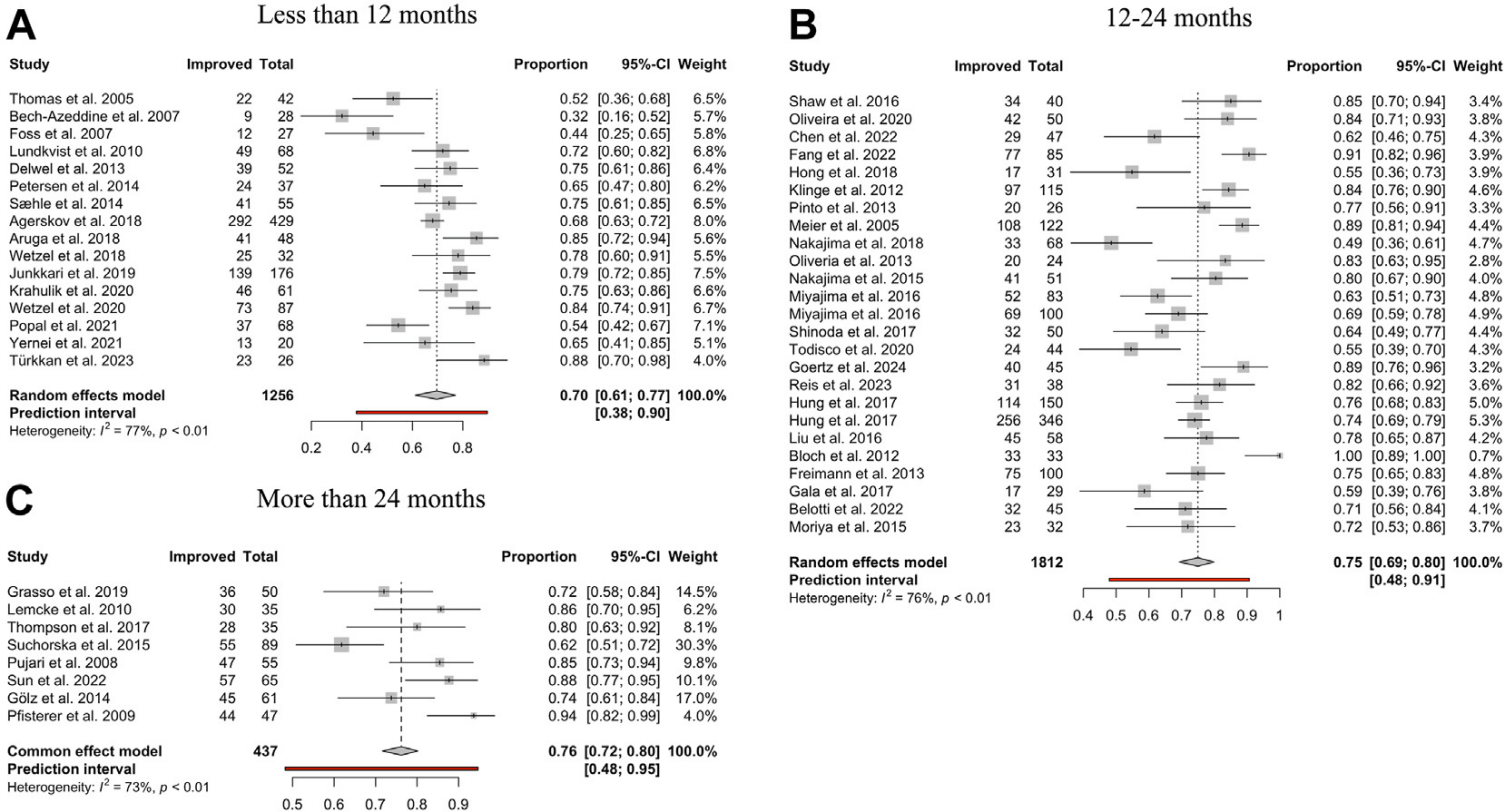
**Shunt response by follow-up period by all shunt procedures. (A–C)** Forest plots visualising the pooled proportion of clinical improvement following shunt procedure when stratified by follow-up study period in the order of A “Less than 12 months”, B “12–24 months”, and C “more than 24 months.” The size of the grey square of the “Proportions” visual correlates to study sample size and the horizontal line indicates the confidence interval. The diamond at the bottom indicates the overall pooled proportion. The red bar below indicates the prediction interval. Heterogeneity is indicated by the chi-squared statistic (I^2^) with associated r^2^ and p-value. The 95% confidence intervals (CI) are shown in squared brackets ([ ]). The weighting of each study is by percentage (%). Furthermore, for every study, the following are displayed: study author with publication date (“Study”), total sample size number for each study (“Total”), and number of clinically improved patients (“Improved”).

Patients with a follow-up period of less than 12 months demonstrated an improvement rate of 70% (95% CI 61–77%), while those with a follow-up duration of 12 months or more but less than or equal to 24 months experienced an improvement rate of 75% (95% CI: 69–80%) following shunt surgery. Studies with a follow-up period of beyond 24 months had an improvement rate of 76% (95% CI: 72–80%) (Fig. 5).

#### Complication profile

41 studies investigated complications after surgery for iNPH. A composite complications endpoint included subdural collections, clinical overdrainage without radiological changes, infections, intracerebral haemorrhage or ischaemic events, shunt malfunction, and mortality. Out of 4099 patients, the overall surgical complications rate was 20.6%. The most common complications were subdural collections in 7.3% (n = 297) and shunt malfunction in 6.0% of cases (n = 246). Out of the subdural collections, most were subdural haematomas (61.3%), followed by subdural hygromas (19.8%), and the rest were unspecific (18.9%). Shunt obstruction accounted for most shunt malfunction cases (69.1%). Clinical signs of overdrainage (e.g., postural headache) without radiological changes occurred in 3.6% of cases (n = 147). The overall infection rate was approximately 2% (n = 70), while haemorrhagic and ischaemic events occurred in less than 1% of patients (n = 36). Mortality due to CSF diversion surgery was uncommon, only occurring in 0.2% of cases (n = 9).

**Fig. 6:**
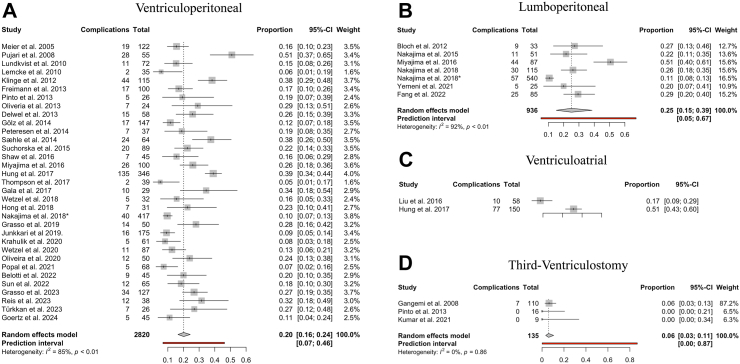
**Complication rate by CSF diversion method. (A–D)** Forest plots visualising the pooled proportional complication rate following CSF diversion in iNPH for each surgical procedure in the order of: “Ventriculoperitoneal”, “Lumboperitoneal”, “Ventriculoatrial” and “Third-Ventriculostomy.” Meta-analysis was not performed for “Ventriculoatrial” due to a low study number (n < 3). The size of the grey square of the “Proportions” visual correlates to the study sample size, and the horizontal line indicates the confidence interval. The diamond at the bottom indicates the overall pooled proportion. The red bar below indicates the prediction interval. Heterogeneity is indicated by the chi-squared statistic (I^2^) with associated r^2^ and p-value. p-value < 0.05 is deemed significant. The 95% confidence intervals (CI) are shown in squared brackets ([ ]). The weighting of each study is by percentage (%).Furthermore, for every study, the following are displayed: study author with publication date (“Study”), total sample size number for each study (“Total”), and number of complications (“Complications”). ∗ = Complications of the nationwide Japanese study,^17^ were detailed in a separate report by Nakajima et al.^73^

VP shunting exhibited a complication rate of 20% (95% CI: 16–24%), with LP shunting at 25% (95% CI: 15–39%). ETV had a low complication rate of 6% (95% CI: 3–11%) (Fig. 6). Meta-analysis for VA shunts was not conducted due to insufficient studies reporting complications (n < 3). There were no notable differences in the complication profiles between the CSF diversion procedures (Supplementary Table S5).

#### Revision surgeries

**Fig. 7:**
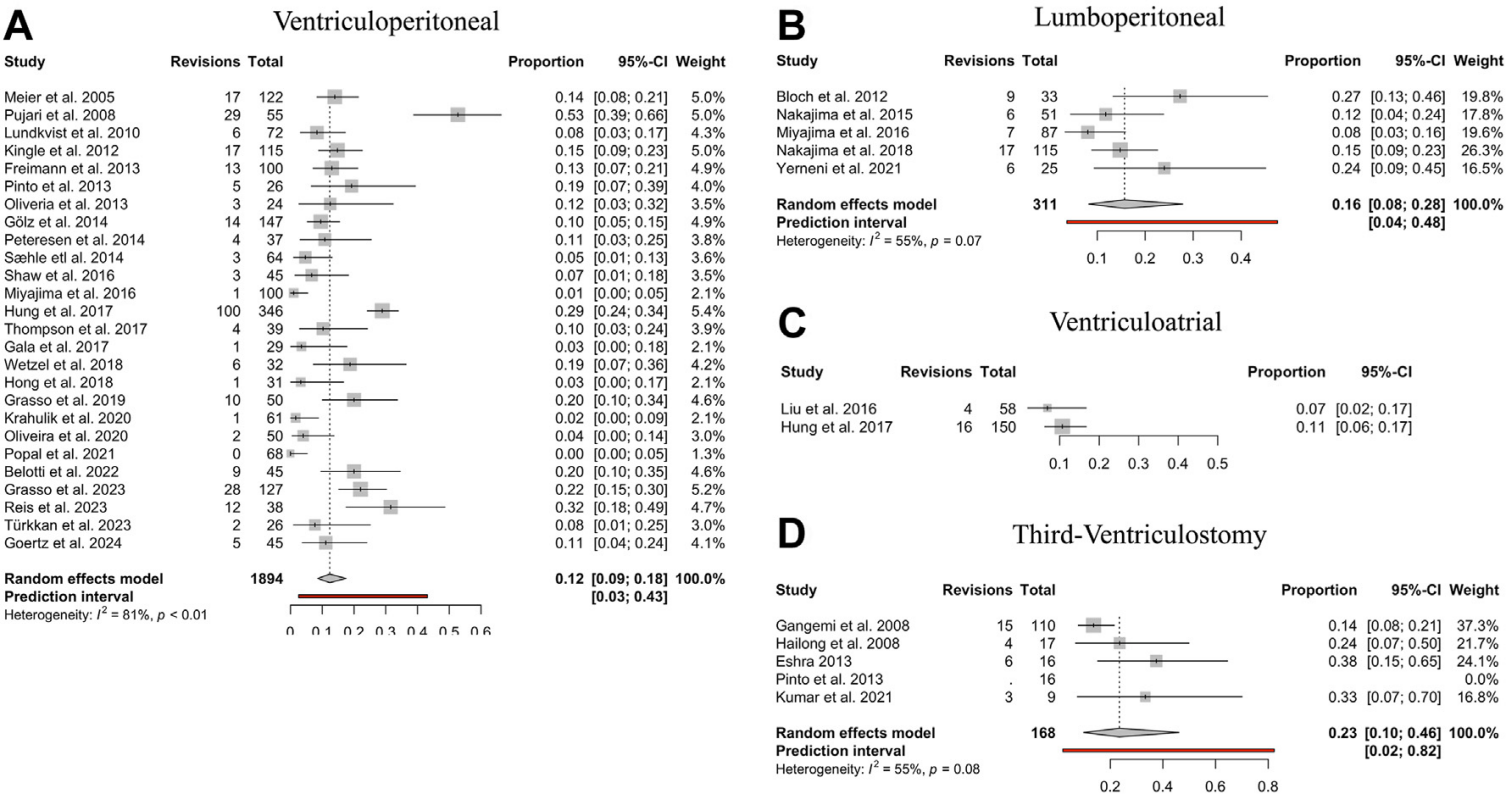
**Surgical revisions by CSF diversion method. (A–D)** Forest plots visualising the pooled proportion of surgical revision rate following CSF diversion in iNPH for each surgical procedure in the order of: “Ventriculoperitoneal”, “Lumboperitoneal”, “Ventriculoatrial”, and “Third-Ventriculostomy.” Meta-analysis was not performed for “Ventriculoatrial” due to the low study number (n < 3). The size of the grey square of the “Proportions” visual correlates to study sample size and the horizontal line indicates the confidence interval. The diamond at the bottom indicates the overall pooled proportion. The red bar below indicates the prediction interval. Heterogeneity is indicated by the chi-squared statistic (I^2^) with associated r^2^ and p-value. p-value < 0.05 is deemed significant. The 95% confidence intervals (CI) are shown in squared brackets ([ ]). The weighting of each study is by percentage (%). Furthermore, for every study the following are displayed: study author with publication date (“Study”), total sample size number for each study (“Total”), and number of surgical revisions (“Revisions”).

34 studies investigated the incidence of surgical revision after the initial CSF diversion procedure. In total, 15.2% of cases necessitated surgery revisions (389 out of 2565 cases). ETV exhibited a revision rate of 23% (95% CI: 10–46%). VP shunting demonstrated a revision rate of 12% (95% CI: 8–17%) (Fig. 7). Meta-analysis for VA shunts was not conducted due to insufficient studies reporting shunt revisions (n < 3).

#### Meta-regression analysis

The meta-regression analysis from Table 2 revealed statistically significant relationships for specific variables affecting the efficacy of CSF diversion surgeries.

**Table 2:** Meta-regression analyses.

	VPS	LPS	VA	Overall Shunt	ETV
**Predictor**					
Age	0.0067 (0.0303)	−0.3939 (0.2667)	N/A	0.0002 (0.0299)	−0.0728 (−0.0728)
Gender (Female)	0.0008 (0.0124)	−0.0541 (0.0461)	−0.0299 (0.0318)	−0.0156 (0.0114)	0.0026 (0.0068)
Hypertension	0.0218 (0.0124)	−0.0141 (0.1495)	N/A	0.0183 (0.0121)	N/A
Diabetes	0.0056 (0.0196)	0.0717 (0.0395)	N/A	0.0218 (0.0170)	N/A
Triad	0.0182 (0.0184)	N/A	N/A	0.0238 (0.0172)	N/A
Gait impairment	0.0052 (0.0081)	0.0421 (0.0483)	0.0083 (0.0025)	0.0071 (0.0065)	0.0386 (0.0996)
Cognitive impairment	0.0086 (0.0079)	−0.0255 (0.0281)	0.0087 (0.003)	0.0037 (0.0065)	0.01 (0.0258)
Urinary incontinence	0.0042 (0.0064)	0.0237 (0.0909)	0.0335 (0.0139)	0.0009 (0.0061)	0.0242 (0.0967)
Symptom duration	0.0188 (0.0188)	N/A	N/A	0.0138 (0.0180)	N/A
Preoperative mRS score	1.6597 (0.9163)	**2.1376 (0.5267) [0.0457]∗**	N/A	**1.2987 (0.4837) [0.0250]∗**	N/A
Preoperative iNPH grading scale score	0.4410 (1.1120)	0.642 (0.3766)	N/A	0.4773 (0.2887)	−1.6967 (1.2289)
Preoperative Evans index	−0.2221 (0.4647)	N/A	N/A	−1.0021 (0.5653)	N/A
Shunt model	0.2897 (0.9131)	2.9629 (1.0774)	N//A	−0.4857 (0.8365)	–
Shunt valve type	0.2675 (0.7671)	**−3.2558 (1.2413) [0.0394] ∗**	−0.1766 (0.4952)	0.4592 (0.6349)	–

The results of the meta-regression of the meta-analyses of VPS, LPS, VA, Overall Shunt and ETV for each covariate as independent variables, to the dependent variable of surgery improvement proportion are shown. In round brackets are the 95% confidence intervals. If the significance is met (denoted with ∗ and a bolded regression coefficient), the p-value of the regression coefficient is shown in a squared bracket; otherwise, assume non-significance. Significance is assumed for p < 0.05. The different explanatory variables were calculated singularly as sole covariates in separate meta-regression analyses. N/A indicates when an insufficient (n < 3) number of studies reported the explanatory variables to perform meta-regression.

VPS: Ventriculoperitoneal shunt. LPS: Lumboperitoneal shunt. VA: Ventriculoatrial. ETV: Endoscopic Third-Ventriculostomy. mRS: modified Ranking Scale. iNPH: idiopathic normal pressure hydrocepahlus.

Notably, a significant positive predictor for overall shunt surgery success was the preoperative modified Rankin Scale (mRS) score with a coefficient of 1.3 (p = 0.025), indicating that a higher mRS score was associated with improved surgical outcomes. A similar significant relationship was observed specifically among studies of LP shunt, with a higher coefficient of 2 with mRS score. Conversely, the use of a fixed-setting valve was a significant negative predictor (p = 0.03).

#### Temporal and sensitivity analysis

**Fig. 8:**
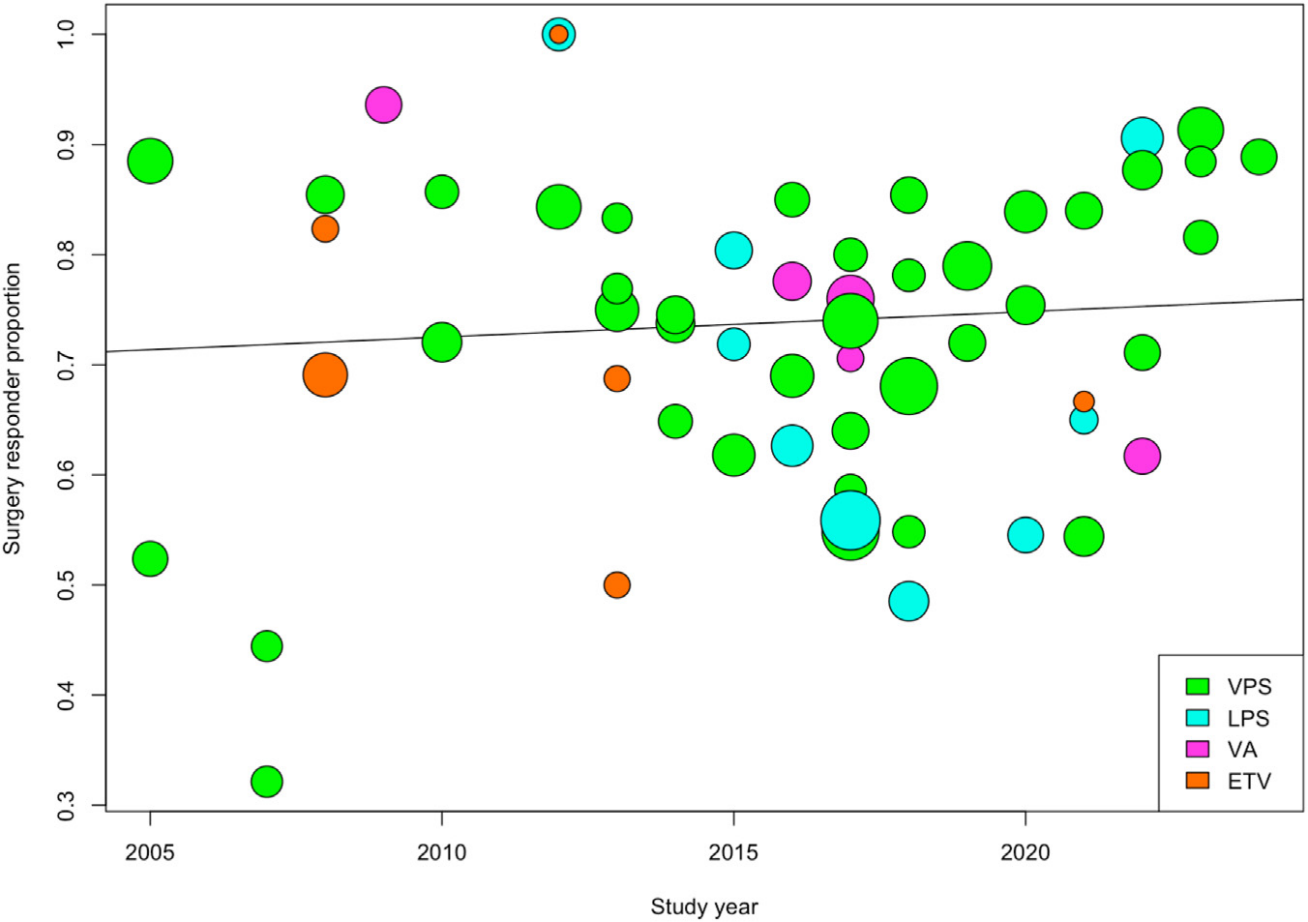
**Temporal trend of the efficacy of CSF diversion in iNPH.** A bubble plot indicating the proportion of surgery responders of all the included studies by publishing year. The x-axis shows the study year, and the y-axis shows the number of surgery responders/divided by the total N number. The size of each bubble indicates study sample size. Each colour represents a different CSF diversion procedure: green for VP shunt, light blue for LP shunt, pink for VA shunt and orange for ETV. A mixed effect meta-regression model line is shown, and no significant trend is found (regression co-efficient = 0.0023, p-value = 0.5420).

The temporal analysis based on Fig. 8 indicates the distribution of overall iNPH surgery success rates over time from 2005 to 2024. The size of each point on the plot corresponds to the study weight. Although varying success rates were observed, there has been no clear trend with time, indicating the year of study was not a significant factor in surgical success (p = 0.54) (Fig. 8). Further stratification by CSF diversion surgery (Supplementary Table S6) confirmed these findings, finding no significant temporal relationship for VP, LPS, and ETV procedures. VA showed a negative trend, indicating a decline in surgical success rates over time (Supplementary Table S6).

The study quality, determined by NOS, did not have a significant impact on the effect size (p = 0.70) (Supplementary Table S6). Pooled data focused on prospective studies alone had an estimated success of 76% (95% CI: 69–82%), which was not significantly different from the estimate derived from only retrospective studies at 74% (95% CI: 69–79%) (p = 0.53) (Supplementary Fig. S3). The exclusion of outlier studies, as identified by the influence analyses, did not significantly alter the effect size, indicating robustness in the meta-analysis (Supplementary Figs. S4–S12).

## Discussion

To our knowledge, this study represents the largest systematic review and meta-analysis of CSF diversion surgery outcomes among iNPH patients to date. By pooling outcomes from 54 studies encompassing 4811 iNPH patients across all major procedures, this analysis provides a detailed evaluation of the effectiveness and safety of surgical treatment in managing iNPH. There was a high improvement rate, which was, however, unchanged over the last 19 years. While heterogeneity between studies and sparse head-to-head data of different CSF diversion surgeries limits direct comparison, our results showed similar efficacy across the procedures.

The 74% overall symptom improvement rate at follow-up indicates that with functioning shunts and careful patient selection, surgical outcomes are generally positive. Earlier reviews on iNPH management reported low rates of improvement after surgery.^74^ This variability is partly historical; Hakim and Adams’ original description focused on standout cases showing dramatic symptomatic reversibility with CSF diversion.^1^ However, as shunting was further studied, it became apparent that clinical responses varied widely.^75^^,^^76^ This variability was often due to suboptimal patient selection, with symptoms of iNPH overlapping with those of neurodegenerative conditions that do not improve with shunting. This study attempts to address this issue by employing iNPH diagnostic criteria in line with consensus statements, ensuring that the patients included had strong clinical suspicion of iNPH, leading to higher validity in the pooled estimates.^12^^,^^13^ The pooled improvement rate from this meta-analysis is essential for informing patients about the potential chance of treatment success, while also providing a benchmark for future iNPH research.

Among the triad of iNPH symptoms, gait ataxia was shown to have high improvement following CSF diversion in this study. Gait abnormalities, such as reduced gait velocity, stride length, and floor clearance are known to improve significantly after shunting,^77^ with previous reports observing early and sustained improvement among 64–77% of cases.^37^^,^^39^ In contrast, cognitive improvements are more variable, with around a 50% improvement rate observed in this meta-analysis, similar to the Study of Idiopathic Normal Pressure Hydrocephalus on Neurological Improvement 2 (SINPHONI-2) trial findings.^6^ This disparity likely stems from the different pathophysiology of these symptoms. Gait symptoms are mainly linked to the frontal subcortical circuits and periventricular white matter, which are affected by ventricular enlargement and therefore are likely to improve with the mechanical CSF decompression achieved by shunting.^78^ Cognitive symptoms, however, involve more extensive brain networks, including the hippocampus, and can overlap with dementia subtypes that are not responsive to shunting.^79^ Urinary symptoms improved in about half of the patients after surgery, likely due to the normalisation of pressure in periventricular white matter tracts involved in bladder control.^80^ However, improvement in urinary symptoms may be less pronounced due to the complex and widespread network involved in bladder control and potential concomitant neurodegenerative changes that are not as reversible.^81^

The sustained improvement in clinical symptoms over extended follow-up periods in this study was somewhat unexpected, given previous findings of a diminishing long-term effect of CSF diversion surgery.^60^^,^^82^ This sustainment may be due to most long-term studies being conducted in large-volume tertiary centres with stringent follow-up protocols that could lead to favourable surgical outcomes. However, up to 20% of patients experience secondary deterioration several years after initial improvement, known as ‘late shunt non-responders’.^83^ This phenomenon is likely due to shunt dysfunction and the progression of neurodegenerative disease, which remains poorly described.^81^ The advancement and wider use of adjustable shunt valves have allowed for the ‘titration’ of optimal CSF flow, addressing secondary deterioration in some individuals.^83^

Our stratified results reveal similar efficacy across different CSF diversion surgeries, with effectiveness generally around 69–75% and overlapping confidence intervals. This was based on improvement proportion, with insufficient head-to-head studies for direct comparisons. Since normal-pressure hydrocephalus was first described in 1965, guidelines have advocated for CSF shunting but no specific surgical procedure.^4^ Nevertheless, VP shunt placement is the most common iNPH treatment modality worldwide.^75^ LP shunts, historically less favoured due to high failure rates and CSF overdrainage, are currently more common in Japan and other regions to minimise the risk of intracranial complications.^45^ Several studies previously reported the safety and non-inferior effectiveness of LP shunts compared to VP shunts, and our analysis supports the notion that we cannot definitively suggest one over the other with similar outcomes observed.^35^^,^^84^ Although, regional biases do exist, with most LP studies originating from East Asia and VP studies conducted in Europe or North America. Different diagnostic thresholds are present, with European studies tending to have a lower average age of iNPH patients.^85^ Additionally, the higher prevalence of obesity and cardiovascular disease comorbidities in Europe and North America impacts shunt success as well as iNPH disease prognosis.^86^ VA shunts, that place the distal catheter in the right cardiac atrium, can be an effective alternative and reduce operative time but are generally only considered when VP shunting is not feasible, such as in patients with abdominal adhesions.^7^ Neurosurgeons tend to avoid VA shunts based on reports of severe haematological and cardiopulmonary complications, although these are largely based on paediatric studies with limited adult data.^4^ A 2017 study by Hung et al. found that among iNPH patients, VA shunts significantly reduced shunt obstruction and surgery revisions compared to VP shunts, with no cardiopulmonary complications observed.^11^ However, more definitive, updated studies are needed to confirm this complication profile. Hence, the ultimate choice of which shunt should be chosen depends on local surgical expertise and patient characteristics until definitive evidence emerges.

ETV involves perforating the third ventricle floor to allow CSF efflux toward the basal cisterns.^5^ Analysis of ETV outcomes among iNPH should be approached with caution due to the limited sample sizes with high heterogeneity between reports. Most studies, aside from Gangemi et al., involved a small number of patients.^58^ Although ETV is a less invasive method of creating a CSF bypass,^87^ recent guidelines do not recommend it due to inconsistent findings.^12^ This review found that ETV offers modest clinical improvements within a wide confidence interval and a high associated surgical revision rate. Another difficulty is the selective anatomical considerations required to undergo endoscopic procedures, with some patients found to be ineligible based on magnetic resonance imaging findings.^45^

While improvement rates after surgery for iNPH are high, the associated adverse events can be severe and vary in frequency.^75^ Subdural haematomas and hygromas were commonly observed complications, that range from asymptomatic radiological findings to medical emergencies. However, most subdural haematomas have been shown to regress spontaneously after increasing valve pressure, with only 10% requiring surgical evacuation.^88^ Our findings suggest mechanical shunt problems, including obstruction and migration, are frequent, and these are known factors contributing to reoperations.^60^ Shunt obstructions are important to treat promptly as approximately 75% of patients can subsequently symptomatically improve.^89^ Early iNPH studies reported surgical complication rates up to 40%,^75^ whereas our review found a significantly lower rate around 20%, consistent with findings from the European multicentre iNPH study.^49^ This reduction can be attributed to advancements in surgical techniques as well as shunt technology including gravitational control devices and improved antibiotic protocols, which have markedly reduced complications associated with CSF overdrainage and shunt-associated infections.^90^ Adjustable valves have also been shown to halve the incidence of subdural collections compared to fixed-pressure valves, supporting their higher costs.^65^

Since the release of the first consensus iNPH guidelines in 2005, surgical success rates have remained consistent, with no significant improvement observed up to 2024 in this review.^75^ This stagnation, reflected by a similar efficacy rate of 71% reported in a 2013 meta-analysis, suggests that outcomes in the management of iNPH may have plateaued.^91^ This could be due to diagnostic challenges, particularly in identifying ‘shunt-responsive’ iNPH patients. Predictive diagnostic methods such as extended lumbar drainage and intracranial pressure monitoring have been found to reliably identify shunt responders.^15^ While operator and centre dependencies may exist, these methods have been successfully replicated and could enhance the current diagnostic process for iNPH.^64^ Interestingly, the meta-regression results showed that patients with greater neurological morbidity, as measured by mRS, experienced greater improvement with shunting, possibly due to more severe symptoms making the classification of shunt responsiveness easier. Additionally, fixed shunt valves were associated with negative outcomes for LP shunt, this impact of valve settings might be more pronounced due to the shunt’s dependency on gravity and patient position compared to VP shunting. The imaging appearance of disproportionately enlarged subarachnoid space hydrocephalus (DESH) has been shown to have a high predictive value for identifying shunt-responsive iNPH patients, however, there were an insufficient number of studies in this review reporting the incidence of DESH.

This study has several limitations. There was significant heterogeneity between individual studies as evidenced by high I^2^ values in the pooled estimates for sym. This may be due to the absence of a standardised outcome assessment for iNPH, resulting in the decision to accept several outcome scales. Additionally, variations in inclusion criteria for shunt insertion, prognostic test usage, shunt valve types, and the use of antisiphon devices further complicate pooling results. The authors utilised sensitivity analysis methods to mitigate this heterogeneity. Additionally, the small number of randomised trials between CSF diversion procedures limits direct comparisons. Most included studies were retrospective however, we found that this did not impact the overall estimate. Lastly, data was collected at the study level; patient-level data would have provided a more detailed insight.

Overall, this study demonstrated a consistently high improvement rate across various CSF diversion surgeries, but significant heterogeneity emphasises the complexity of studying iNPH management. This analysis supports the notion that the choice between VP, LP and VA shunts should ultimately be based on the surgeon’s preference and the patient’s medical history. Despite positive outcomes, the stagnation in success rates over the past two decades highlights a research need for improved patient selection and the potential benefit of advanced diagnostic techniques in clinical practice.

## Contributors

A.S and S.G.T accessed and verified all the data in the study and take responsibility for the integrity of the data and the accuracy of the data analysis. A.S. and S.G.T. were involved in conceptualization, data curation, formal analysis, investigation, methodology, project administration, software, supervision, validation, visualization, writing—original draft, and writing—review and editing. A.A and M.V were involved in data curation, formal analysis, investigation, validation and writing—original draft. R.T.F, A.K, D.K, M.O., D.J. were involved in conceptualization, writing—original draft, and writing—review and editing. F.R. was involved in conceptualization, formal analysis, investigation, supervision, validation, visualization, writing—original draft, and writing—review and editing.

## Data sharing statement

All data used for the study has been included in the manuscript and Supplementary material.

## Declaration of interests

Santhosh G. Thavarajasingam receives consultation payments from Brainlab. Florian Ringel receives payments from Stryker, Spineart, Brainlab. All data and materials as well as software application support their published claims and comply with field standards.
