# Supplementary material for: The effectiveness of various CSF diversion surgeries in idiopathic normal pressure hydrocephalus: a systematic review and meta-analysis
Source: eClinicalMedicine. 2024 Oct 30;77:102891. doi: 10.1016/j.eclinm.2024.102891 (PMC11558045; doi:10.1016/j.eclinm.2024.102891)
Supplement: Supplementary Figs. S1–S12 and Tables S1–S6 [file mmc1.docx]

**The Effectiveness of Various Cerebrospinal Fluid Diversion Surgeries in Idiopathic Normal Pressure Hydrocephalus: A Systematic Review and Meta-Analysis**

**Supplementary Appendix**

Ahmed Salih^1,2^; Aksaan Arif ^1,2^

Madhur Varadpande^1,2^ ; Rafael Tiza Fernandes^2,3^

Dragan Jankovic^4^ ; Darius Kalasauskas^4^

Malte Ottenhausen^4^ ; Andreas Kramer^4^

Florian Ringel^4^ ; Santhosh G. Thavarajasingam^2,4 *^

**^INSTITUTION:^**

1. School of Medicine, Imperial College London, London, United Kingdom.
2. Imperial Brain and Spine Initiative, Imperial College London, London, United Kingdom
3. Department of Neurosurgery, ULS São José, Lisbon, Portugal
4. Department of Neurosurgery, University Medical Center Mainz, Mainz, Germany

**Contents**

Supplementary Table 1......................................................................................................................3

Supplementary Table 2 .....................................................................................................................4

Supplementary Table 3......................................................................................................................4

Supplementary Table 4......................................................................................................................5

Supplementary Table 5......................................................................................................................5

Supplementary Table 6......................................................................................................................6

Supplementary Figure 1.....................................................................................................................7

Supplementary Figure 2.....................................................................................................................8

Supplementary Figure 3.....................................................................................................................9

Supplementary Figure 4..................................................................................................................10-11

Supplementary Figure 5..................................................................................................................12-13

Supplementary Figure 6..................................................................................................................13-14

Supplementary Figure 7..................................................................................................................15-16

Supplementary Figure 8..................................................................................................................17-18

Supplementary Figure 9..................................................................................................................19-20

Supplementary Figure 10................................................................................................................21-22

Supplementary Figure 11................................................................................................................24-25

Supplementary Figure 12................................................................................................................26-27

**Supplementary Table 1 – Search strategy**

| Database | Search terms |
| --- | --- |
| Medline | 1 idiopathic normal pressure hydrocephalus.mp.  2 iNPH.mp.  3 idiopathic normotensive hydrocephalus.mp.  4 surg*.mp.  5 shunt*.mp.  6 Cerebrospinal Fluid Shunts/ or cerebrospinal fluid shunt*.mp.  7 intervention*.mp.  8 lumboperitoneal.mp.  9 ventriculoatrial.mp.  10 Ventriculoperitoneal Shunt/ or ventriculoperitoneal.mp.  11 CSF shunt*.mp.  12 ventriculopleural.mp.  13 endoscopic third ventriculostomy.mp. or Ventriculostomy/  14 cerebrospinal fluid diversion.mp.  15 1 or 2 or 3  16 4 or 5 or 6 or 7 or 8 or 9 or 10 or 11 or 12 or 13 or 14  17 15 and 16 |
| Embase | 1 idiopathic normal pressure hydrocephalus.mp.  2 iNPH.mp.  3 idiopathic normotensive hydrocephalus.mp.  4 surg*.mp.  5 shunt*.mp.  6 Cerebrospinal Fluid Shunts/ or cerebrospinal fluid shunt*.mp.  7 intervention*.mp.  8 lumboperitoneal.mp.  9 ventriculoatrial.mp.  10 Ventriculoperitoneal Shunt/ or ventriculoperitoneal.mp.  11 CSF shunt*.mp.  12 ventriculopleural.mp.  13 endoscopic third ventriculostomy.mp. or Ventriculostomy/  14 cerebrospinal fluid diversion.mp.  15 1 or 2 or 3  16 4 or 5 or 6 or 7 or 8 or 9 or 10 or 11 or 12 or 13 or 14  17 15 and 16 |
| Scopus | ( "Idiopathic normal pressure hydrocephalus" OR "iNPH" OR "Idiopathic normotensive hydrocephalus" ) AND TITLE-ABS-KEY ( "surg*" OR "shunt*" OR "cerebrospinal fluid shunt*" OR "intervention*" OR "lumboperitoneal" OR "ventriculopleural" OR "endoscopic third ventriculostomy" OR "ventriculoatrial" OR "ventriculoperitoneal" OR "CSF shunt*" OR "ventriculostomy" OR "cerebrospinal fluid diversion" ) ) |
| Web of science | **idiopathic normal pressure hydrocephalus OR iNPH or idiopathic normotensive hydrocephalus** (All Fields) and **"surg*" OR "shunt*" OR "cerebrospinal fluid shunt*" OR "intervention*" OR "lumboperitoneal" OR "endoscopic third ventriculostomy" OR "ventriculoatrial" OR "ventriculoperitoneal" OR “ventriculopleural” OR "CSF shunt*" OR "ventriculostomy" OR "cerebrospinal fluid diversion"** |

For the Cochrane Library of Registered Clinical Trials and World Health Organisation International Clinical Trials Registry Platform, MESH terms and key terms searching were utilised.

**Supplementary Table 2 – iNPH overall and triad outcome scale assessments**

| **Overall** | **Gait** | **Cognition** | **Urinary** |
| --- | --- | --- | --- |
| NPH grading scale OR iNPHGS (idiopathic normal pressure hydrocephalus grading scale) OR JNPHGS (Japanese Committee for Scientific Research on Intractable Hydrocephalus) grading scale  Modified Rankin Scale (mRS)  Kiefer Score  Black Scale  Quality of Life Scale (EQ-5D-5L)  Steins and Lang Fitts | Ten-meter Walk Test (10MWT)  Timed Up & Go (TUG)  6-Minute Walk Test (6MWT)  Tinetti scale  Gait speed (meters/second) | MMSE (Mini-Mental State Examination)  Improvement in verbal IQ, performance IQ, or full-scale IQ on the Wechsler Adult Intelligence Scale–Revised WAIS-R)  Addenbrooke's Cognitive Examination-Revised  (ACE-R)  Montreal Cognitive Assessment (MOCA)  Frontal Assessment Battery | International Consultation on Incontinence Questionnaire  Reduction of episodes of incontinence or improvement of bladder control  Urinary Incontinence Short Form (ICIq-UI)  The overactive bladder questionnaire (ICIq-OAB)    LUTS QOL  questionnaire (ICIq-LUTSqol) |

**Supplementary Table 3 – Complications of CSF diversion procedures**

| **Complications** |
| --- |
| Shunt malfunction  Infection  Subdural collections (including subdural haematoma, hygroma and unspecified collections)  Clinical signs of overdrainage without radiological changes (including postural headache, dizziness, nausea)  Intracerebral haemorrhagic events  Intracerebral ischaemic events  Mortality |

**Supplementary Table 4: Adapted Newcastle Ottawa Scale**

| **Selection criteria** | Representation of exposure cohort (iNPH patients) (1) |
| --- | --- |
|  | Selection of non-exposure cohort (if applicable) (1) |
|  | Ascertain of exposure (1) |
|  | Demonstration that outcome change over time, absence of presence at beginning (1) |
| **Comparability** | Study design (2 for controlled trial, 1 for prospective, 0 for retrospective) (2) |
| **Outcome** | Assessment of outcome (1) |
|  | Follow-up (if minimum 1 year) (1) |
|  | Adequacy of follow up (>80%) (1) |

**Supplementary Table 5: Specific complication rates for CSF diversion surgeries**

|  | VPS | LPS | VA | ETV |
| --- | --- | --- | --- | --- |
| ***Complications*** |  |  |  |  |
| Number of studies | 32 | 7 | 2 | 3 |
| Subdural haematoma/hygroma | 6.1 (4.4-8.4) | 4.6 (3.3-6.4) | 5-19* | 2.3 (0.7-7.3) |
| Infection | 2.6 (2.1-3.2) | 1.8 (0.8-3.9) | 2-8* | NA |
| Clinical signs of overdrainage without radiological changes | 7.0 (4.0-11.9) | 10.4 (4.8-21.3) | 5-11* | NA |
| Shunt malfunction | 5.3 (3.6-7.8) | 7.5 (3.8-14.1) | 12-23* | NA |
| Intracerebral infarction/haemorrhage | 2.4 (1.0-5.8) | 5.0 (2.6-9.3) | 0-6* | 2.3 (0.7-7.3) |
| Mortality | 2.1 (1.4-3.1) | 0.9 (0.1-5.9) | NA | NA |

The pooled estimated with associated standard deviations in brackets apart. ‘Clinical signs of overdrainage without radiological changes’ include symptoms such as postural headache, dizziness and nausea. Pooled analysis was conducted for all procedures apart from VA due to insufficient number of studies, instead the table shows the range of complications among the included studies (*). NA indicates no data was available.

*VPS; ventriculoperitoneal shunt, LPS; lumboperitoneal shunt, VA; ventriculoatrial shunt, ETV; endoscopic third-ventriculostomy*

**Supplementary Table 6: Meta-regression results**

|  | VPS | LPS | VA | ETV | Overall |
| --- | --- | --- | --- | --- | --- |
| ***Variable*** |  |  |  |  |  |
| Year of study | 0.0402  p-value = 0.068 | -0.0516  p-value = 0.708 | -0.1561  p-value = 0.006 | -0.0345  p-value = 0.570 | 0.0023  p-value = 0.542 |
| Risk of Bias score (low, some concern or high) | - | - | - | - | 0.3623  p-value = 0.700 |


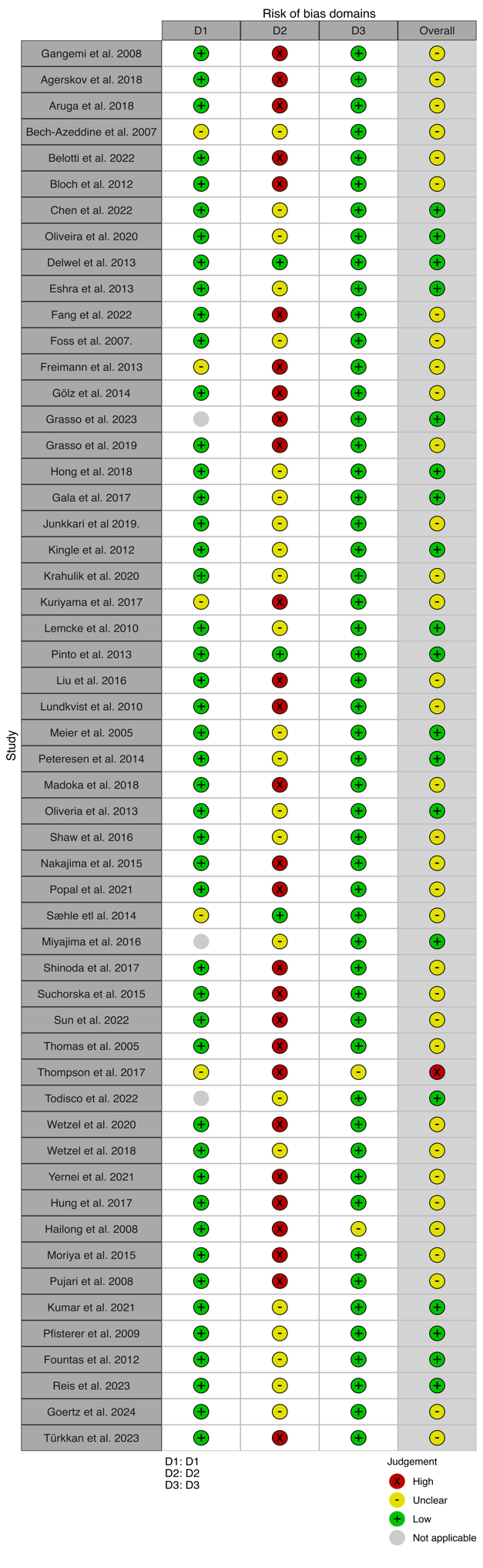

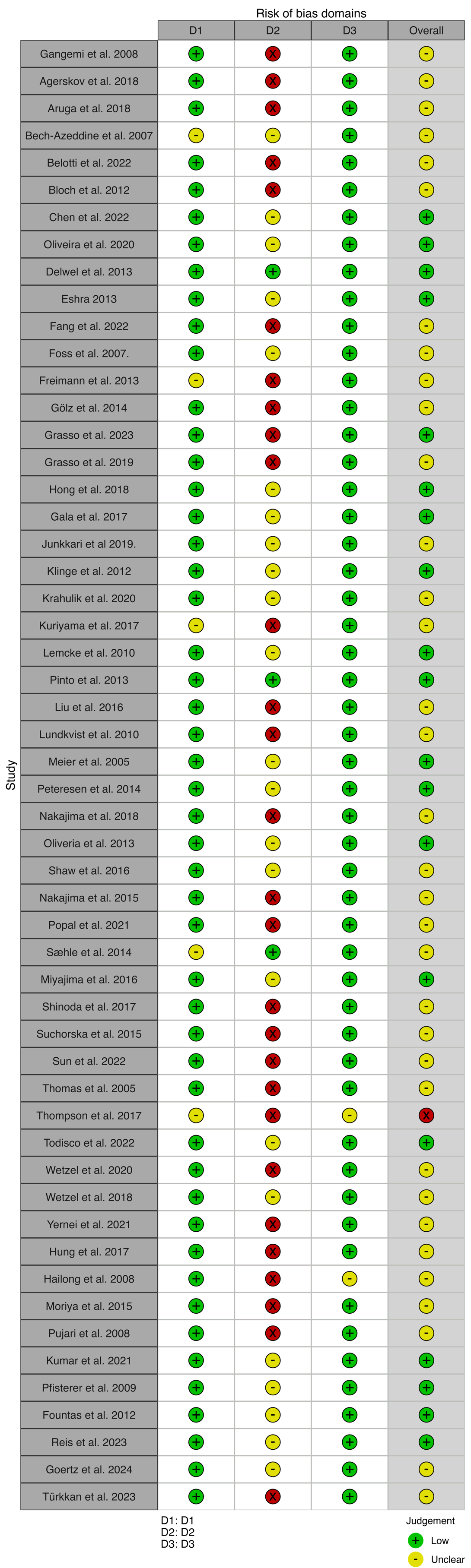


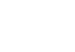
**Supplementary Figure 1**: **Risk of bias assessment using Newcastle-Ottawa Score**

Scores were assigned for bias according to selection criteria (D1), comparability (D2) and outcome (D3) with an overall score out of 9. Risk of bias was deemed as ‘low’ (green), ‘some concerns’ (yellow) or ‘high’ (red) for each domain. A composite NOS score was calculated.


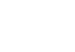
**Supplementary Figure 2: Funnel plot for individual CSF diversion surgery**

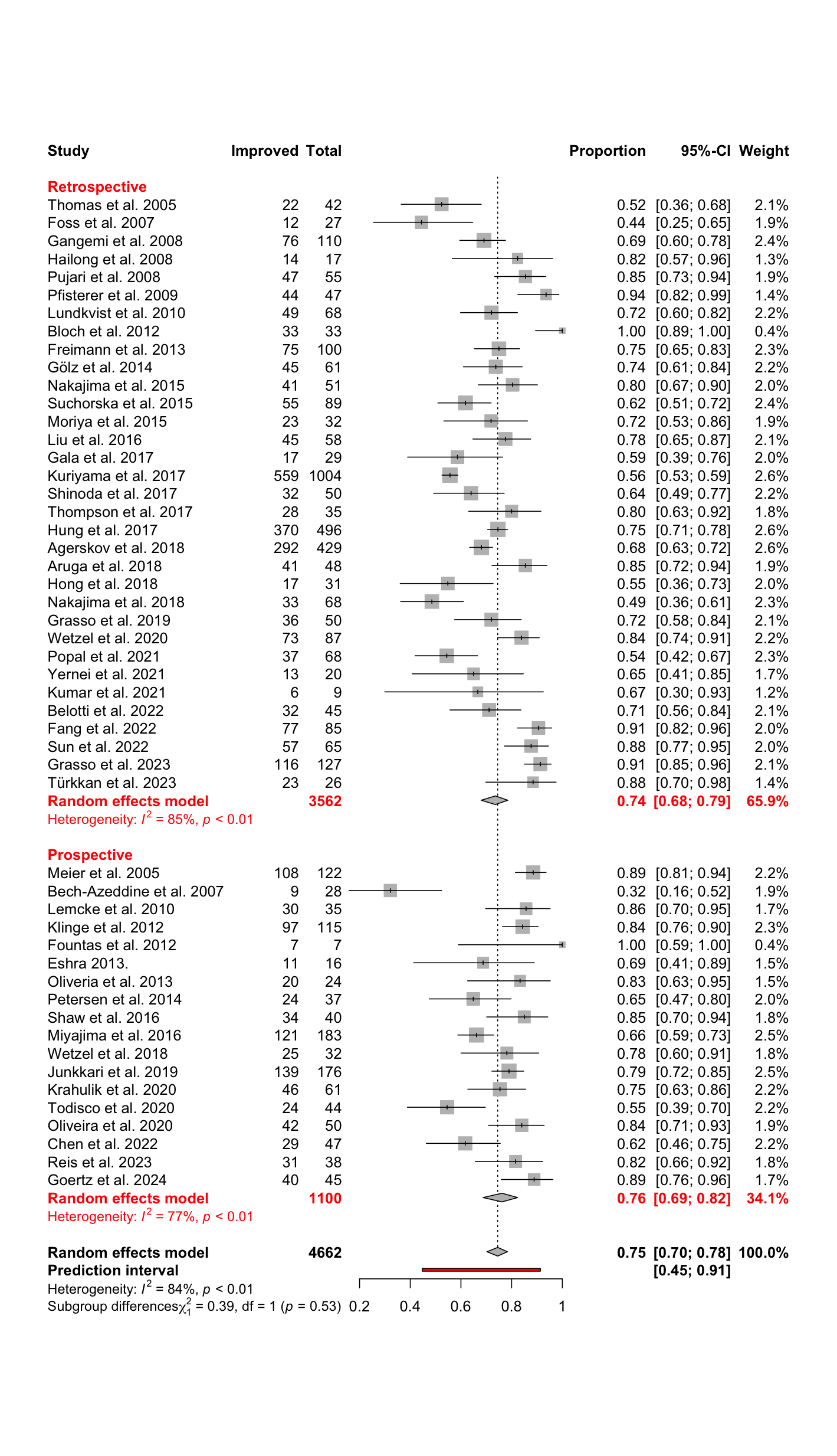
Supplementary Figure 3: **Symptomatic improvement by study design**

**Supplementary Figure 4: Influence analysis for VP shunt and Improvement**

In

Supplementary Figure 4, an influence analysis for VP shunt and improvement*,* is shown in four diagnostic graphs (Supplementary Figure 4A-C). The influence analysis influence analysis aids in identifying studies that contribute highly to the between-study heterogeneity found in the meta-analysis (e.g., outliers) and could therefore be excluded in a sensitivity analysis or have a significant impact on the pooled effect size of a meta-analysis, meaning that the overall effect size may change considerably when the respective study is removed. Supplementary Figure 4A shows a Baujat plot which maps the overall heterogeneity contribution against the influence on pooled results. The studies are denoted as blue circles, with the circle size corresponding to the overall effect on either parameter. Supplementary Figure 4B shows an Influence Characteristics plot, which includes several influence analysis diagnostics. The studies, determined to be skewing factors, using the "rules of thumb" are shown in red. Supplementary Figure 4C shows a forest plot for the leave-one-out analysis, sorted by effect size and heterogeneity. The graph displays the effect size and *I^2^*-heterogeneity when omitting one of the included studies each time. The plot is stratified by effect size to determine which studies or effect sizes particularly affect the overall effect size, towards both extremes. The area shaded green indicates lower impact, if results fall outside, it indicates higher impact.


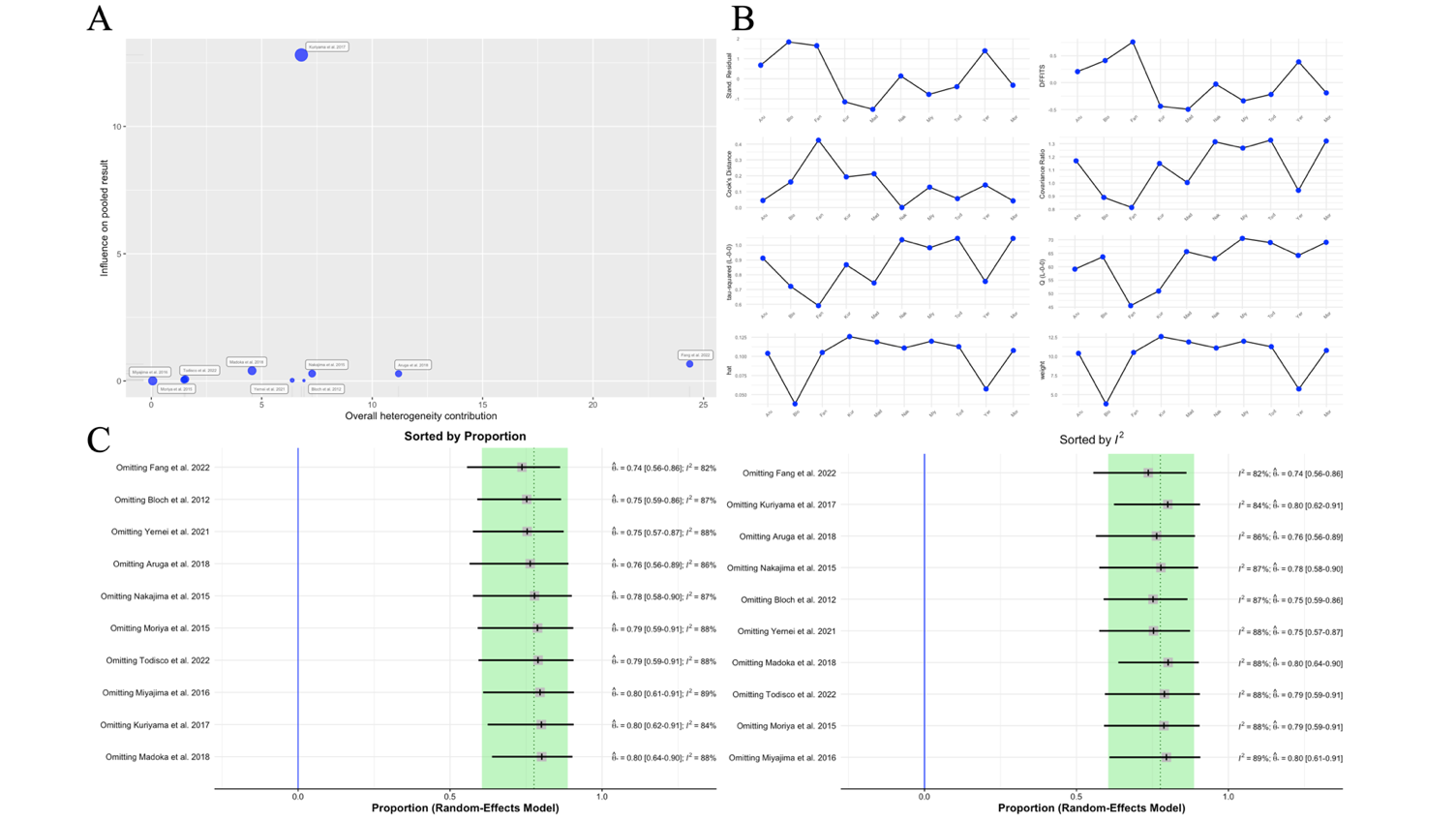
Supplementary Figure 5: **Influence analysis for LP shunt and Improvement**

In Supplementary Figure 5, an influence analysis for LP shunt and improvement is shown in four different diagnostic graphs (Supplementary Figure 5A-C). The influence analysis aids in identifying studies that contribute highly to the between-study heterogeneity found in the meta-analysis (e.g., outliers) and could therefore be excluded in a sensitivity analysis or have a large impact on the pooled effect size of a meta-analysis, meaning that the overall effect size may change considerably when the respective study is removed. Supplementary Figure 5A shows a Baujat plot which maps the overall heterogeneity contribution against the influence on pooled results. The studies are denoted as blue circles, with circle size corresponding to overall effect on either parameter. Supplementary Figure 5B shows an Influence Characteristics plot, which includes several influence analysis diagnostics. The studies, determined to be skewing factors, using the "rules of thumb" are shown in red. Supplementary Figure 3C shows a forest plot for the leave-one-out analysis, sorted by effect size and heterogeneity. The graph displays the effect size and *I^2^*-heterogeneity when omitting one of the included studies each time. The plot is stratified by effect size to determine which studies or effect sizes particularly affect the overall effect size, towards both extremes. The area shaded green indicates lower impact. If results fall outside, it indicates higher impact.

**Supplementary Figure 6: Influence analysis for VA shunt and Improvement**


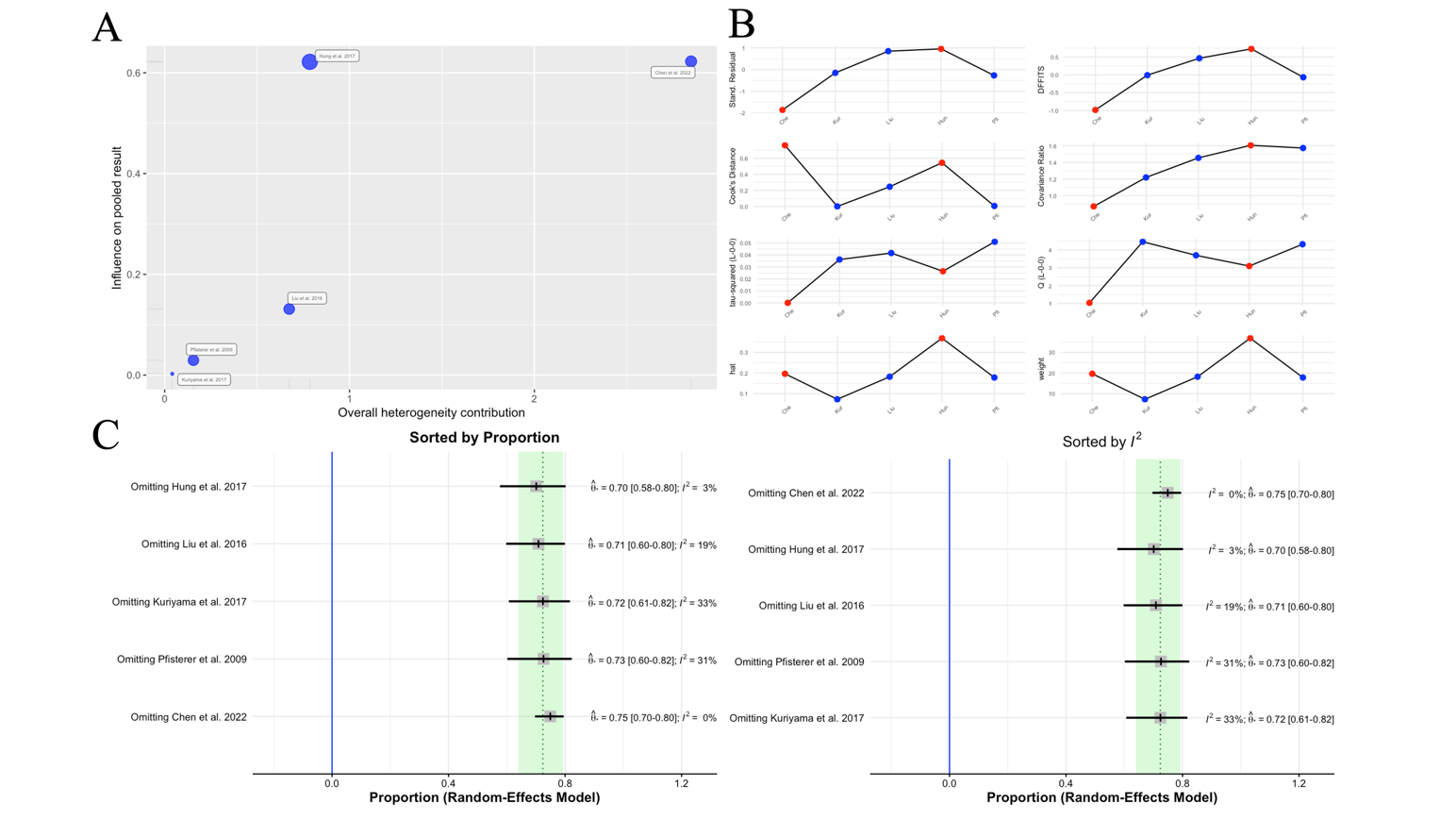


In Supplementary Figure 6, an influence analysis for VA shunt and improvement*,* is shown in four different diagnostic graphs (Supplementary Figure 6A-C). The influence analysis aids in identifying studies that contribute highly to the between-study heterogeneity found in the meta-analysis (e.g., outliers) and could therefore be excluded in a sensitivity analysis or have a large impact on the pooled effect size of a meta-analysis, meaning that the overall effect size may change considerably when the respective study is removed. Supplementary Figure 6A shows a Baujat plot which maps the overall heterogeneity contribution against the influence on pooled results. The studies are denoted as blue circles, with circle size corresponding to overall effect on either parameter. Supplementary Figure 6B shows an Influence Characteristics plot, which includes several influence analysis diagnostics. The studies, determined to be skewing factors, using the "rules of thumb" are shown in red. Supplementary Figure 6C shows a forest plot for the leave-one-out analysis, sorted by effect size and heterogeneity. The graph displays the effect size and *I^2^*-heterogeneity when omitting one of the included studies each time. The plot is stratified by effect size to determine which studies or effect sizes particularly affect the overall effect size, towards both extremes. The area shaded green indicates lower impact. If results fall outside, it indicates higher impact.

**Supplementary Figure 7: Influence analysis for ETV and improvement**


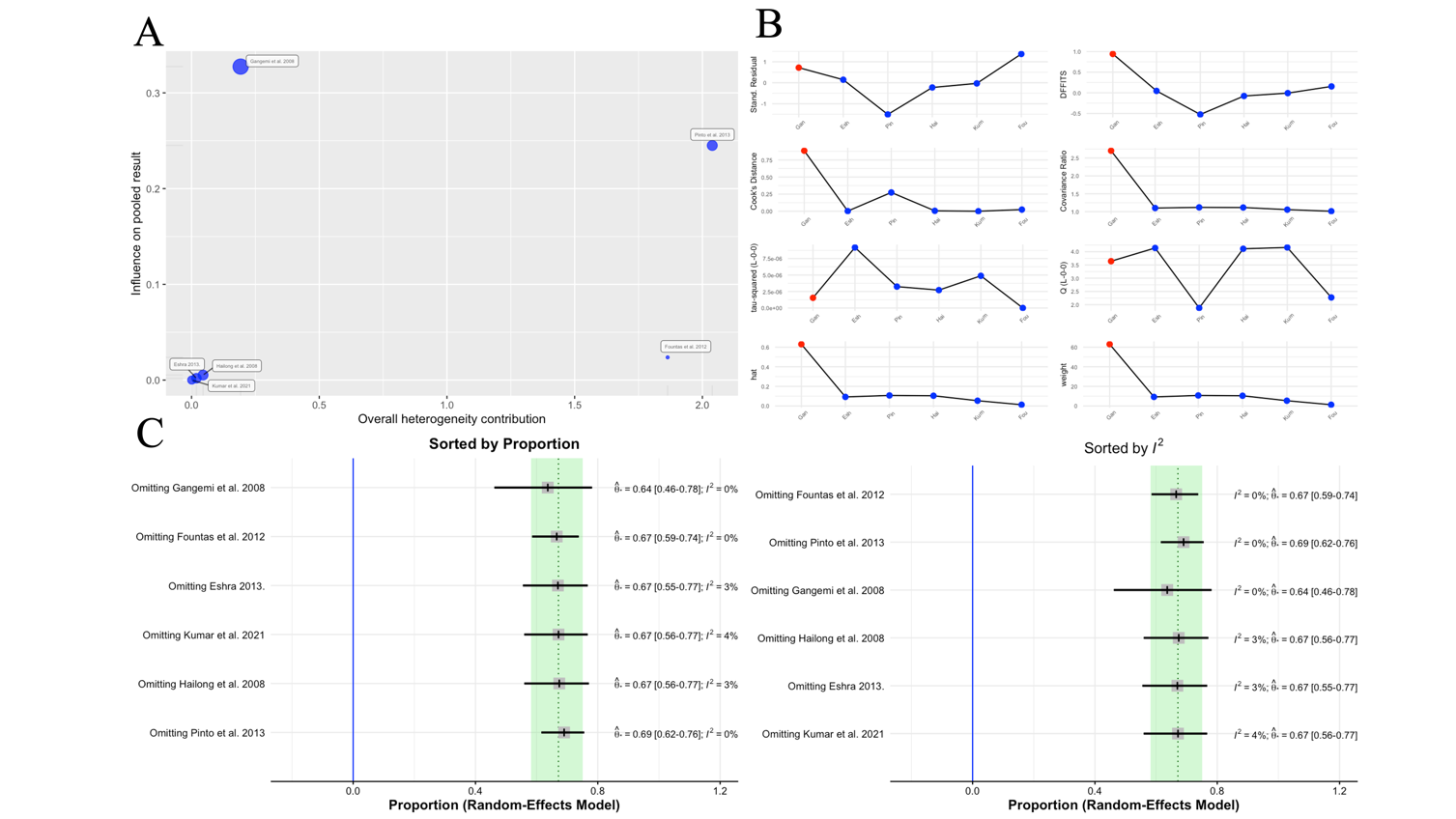


In Supplementary Figure 7, an influence analysis for ETV and improvement*,* is shown in four different diagnostic graphs (Supplementary Figure 7A-C). The influence analysis aids in identifying studies that contribute highly to the between-study heterogeneity found in the meta-analysis (e.g., outliers) and could therefore be excluded in a sensitivity analysis or have a large impact on the pooled effect size of a meta-analysis, meaning that the overall effect size may change considerably when the respective study is removed. Supplementary Figure 7A shows a Baujat plot which maps the overall heterogeneity contribution against the influence on pooled results. The studies are denoted as blue circles, with circle size corresponding to overall effect on either parameter. Supplementary Figure 7B shows an Influence Characteristics plot, which includes several influence analysis diagnostics. The studies, determined to be skewing factors, using the "rules of thumb" are shown in red. Supplementary Figure 7C shows a forest plot for the leave-one-out analysis, sorted by effect size and heterogeneity. The graph displays the effect size and *I^2^*-heterogeneity when omitting one of the included studies each time. The plot is stratified by effect size to determine which studies or effect sizes particularly affect the overall effect size, towards both extremes. The area shaded green indicates lower impact. If results fall outside, it indicates higher impact.

**Supplementary Figure 8: Influence analysis for VP shunt and complications**

In

Supplementary Figure 8, an influence analysis for VP shunt and complications*,* is shown in four different diagnostic graphs (Supplementary Figure 8A-C). The influence analysis aids in identifying studies that contribute highly to the between-study heterogeneity found in the meta-analysis (e.g., outliers) and could therefore be excluded in a sensitivity analysis or have a large impact on the pooled effect size of a meta-analysis, meaning that the overall effect size may change considerably when the respective study is removed. Supplementary Figure 8A shows a Baujat plot which maps the overall heterogeneity contribution against the influence on pooled results. The studies are denoted as blue circles, with circle size corresponding to overall effect on either parameter. Supplementary Figure 8B shows an Influence Characteristics plot, which includes several influence analysis diagnostics. The studies, determined to be skewing factors, using the "rules of thumb" are shown in red. Supplementary Figure 8C shows a forest plot for the leave-one-out analysis, sorted by effect size and heterogeneity. The graph displays the effect size and *I^2^*-heterogeneity when omitting one of the included studies each time. The plot is stratified by effect size to determine which studies or effect sizes particularly affect the overall effect size, towards both extremes. The area shaded green indicates lower impact. If results fall outside, it indicates higher impact.

**Supplementary Figure 9: Influence analysis for LP shunt and complications**


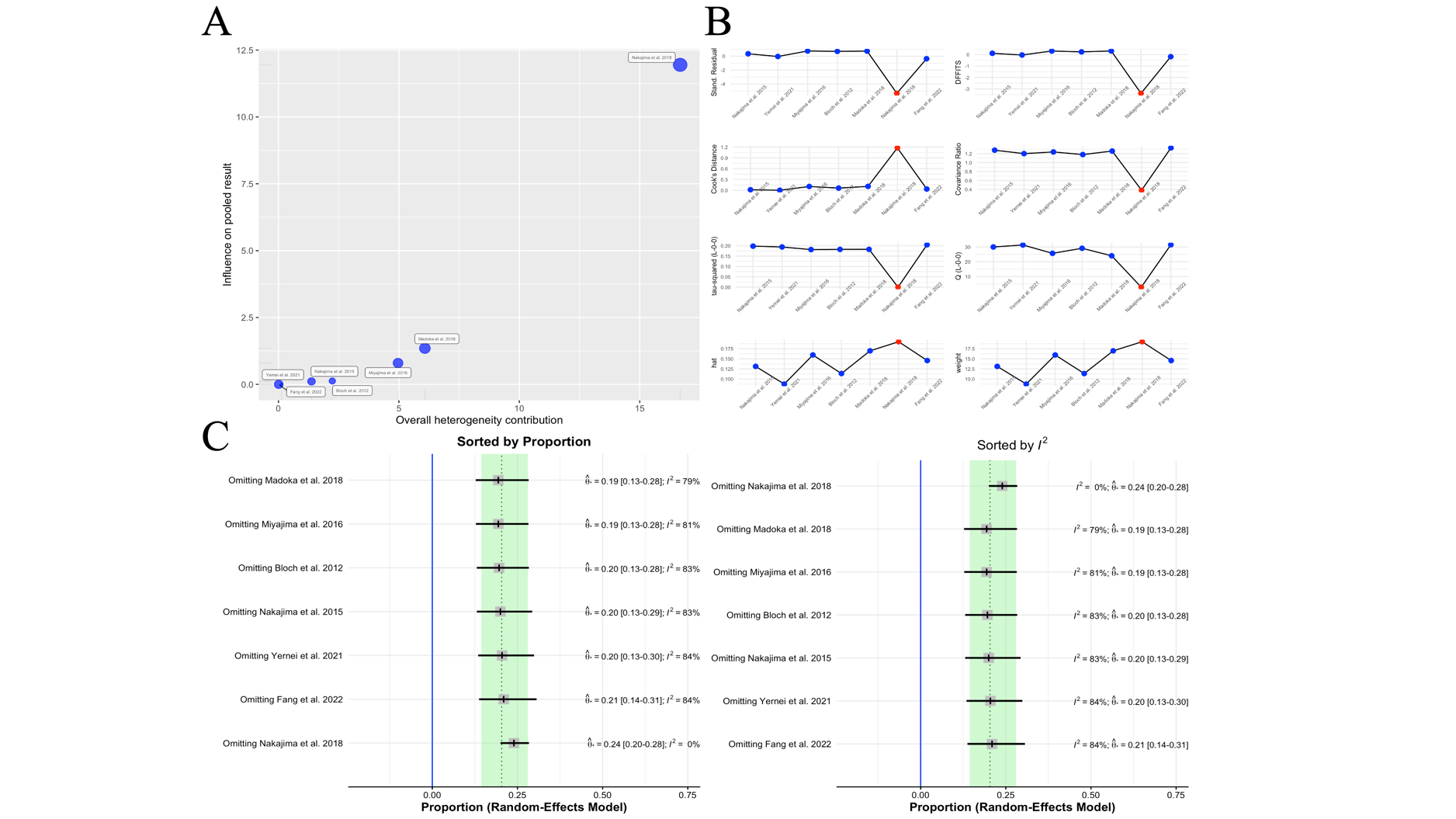


In Supplementary Figure 9, an influence analysis for VP shunt and complications*,* is shown in four different diagnostic graphs (Supplementary Figure 9A-C). The influence analysis influence analysis aids in identifying studies that contribute highly to the between-study heterogeneity found in the meta-analysis (e.g., outliers) and could therefore be excluded in a sensitivity analysis or have a large impact on the pooled effect size of a meta-analysis, meaning that the overall effect size may change considerably when the respective study is removed. Supplementary Figure 9A shows a Baujat plot which maps the overall heterogeneity contribution against the influence on pooled results. The studies are denoted as blue circles, with circle size corresponding to overall effect on either parameter. Supplementary Figure 9B shows an Influence Characteristics plot, which includes several influence analysis diagnostics. The studies, determined to be skewing factors, using the "rules of thumb" are shown in red. Supplementary Figure 9C shows a forest plot for the leave-one-out analysis, sorted by effect size and heterogeneity. The graph displays the effect size and *I^2^*-heterogeneity when omitting one of the included studies each time. The plot is stratified by effect size to determine which studies or effect sizes particularly affect the overall effect size, towards both extremes. The area shaded green indicates lower impact. If results fall outside, it indicates higher impact.

**Supplementary Figure 10: Influence analysis for ETV and complications**


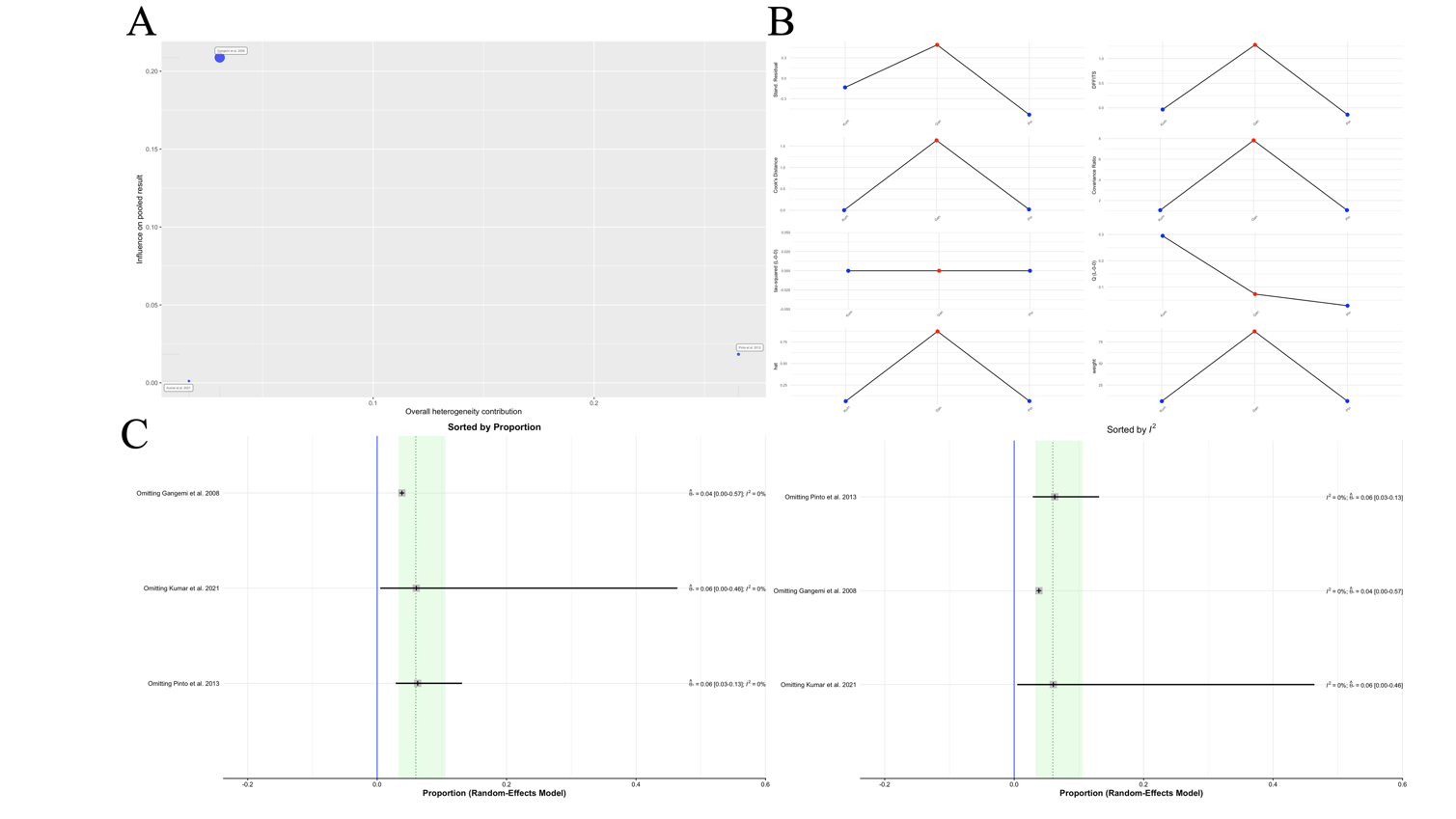


In Supplementary Figure 10, an influence analysis for VP shunt and complications*,* is shown in four different diagnostic graphs (Supplementary Figure 10A-C). The influence analysis aids in identifying studies that contribute highly to the between-study heterogeneity found in the meta-analysis (e.g., outliers) and could therefore be excluded in a sensitivity analysis or have a large impact on the pooled effect size of a meta-analysis, meaning that the overall effect size may change considerably when the respective study is removed. Supplementary Figure 10A shows a Baujat plot which maps the overall heterogeneity contribution against the influence on pooled results. The studies are denoted as blue circles, with circle size corresponding to overall effect on either parameter. Supplementary Figure 10B shows an Influence Characteristics plot, which includes several influence analysis diagnostics. The studies, determined to be skewing factors, using the "rules of thumb" are shown in red. Supplementary Figure 10C shows a forest plot for the leave-one-out analysis, sorted by effect size and heterogeneity. The graph displays the effect size and *I^2^*-heterogeneity when omitting one of the included studies each time. The plot is stratified by effect size to determine which studies or effect sizes particularly affect the overall effect size, towards both extremes. The area shaded green indicates lower impact. If results fall outside, it indicates higher impact.

**
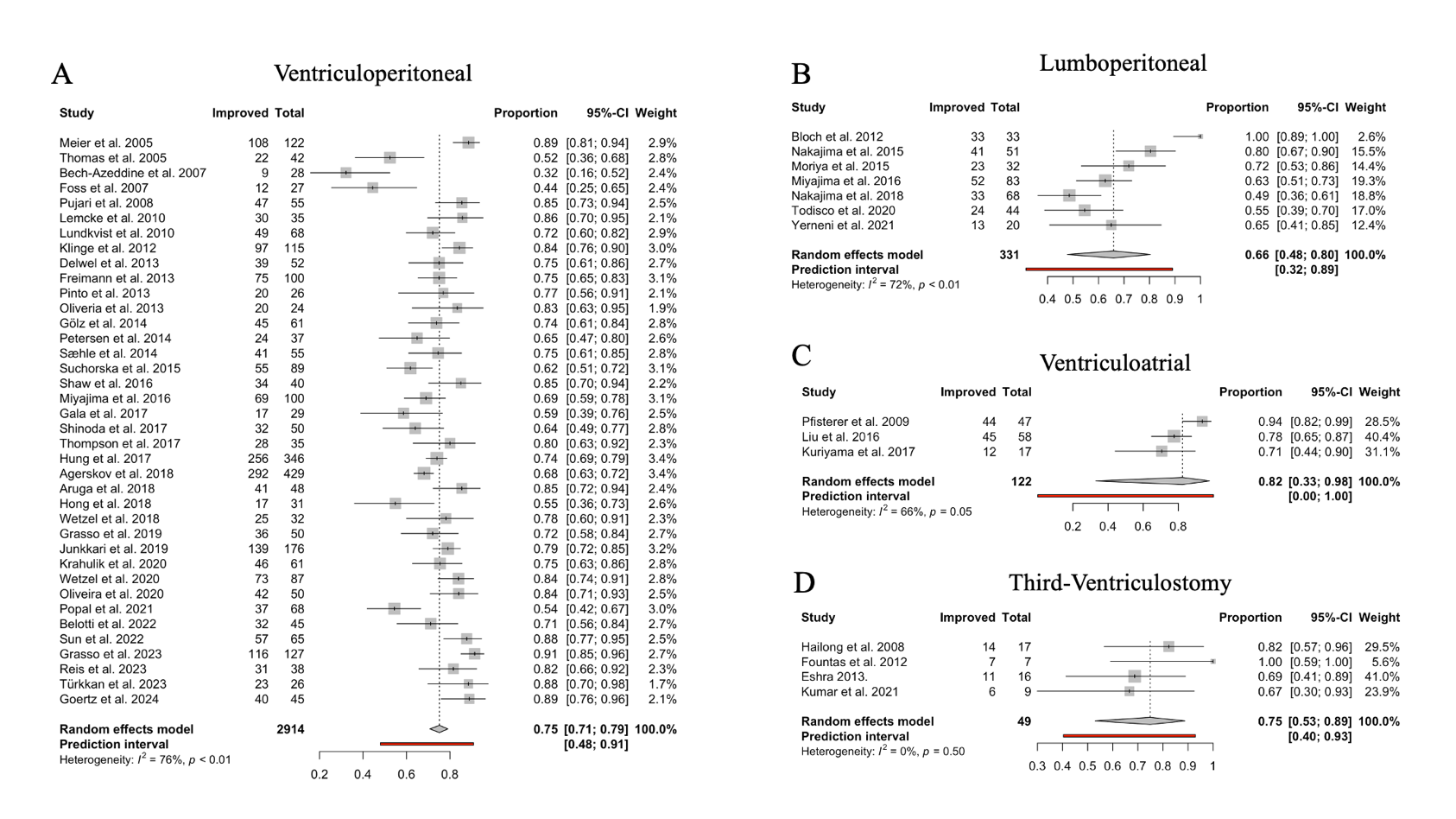
Supplementary Figure 11: Symptomatic improvement excluding outliers**

(A-D) Forest plots visualising the pooled proportional improvement of iNPH patients following CSF diversion for each surgical procedure in the order of: “Ventriculoperitoneal”, “Lumboperitoneal”, “Ventriculoatrial” and “Third-Ventriculostomy.” Excluded studies identified by the influence analysis as outliers were: Junkkari et al. 2019 and Kuriyama et al. 2017, Fang et al. 2022 and Kuriyama et al. 2017, Hung et al. 2017 and Chen et al. 2022 and Gangemi et al. 2008 and Pinto et al. 2013. The size of the grey square of the “Proportions” visual correlates to study sample size and the horizontal lines indicates the confidence interval. The diamond at the bottom indicates the overall pooled proportion. The red bar below it indicates the prediction interval. Heterogeneity is indicated by the chi-squared statistic (I^2^) with associated r^2^ and p-value. p-value < 0.05 is deemed significant. The 95% confidence intervals (CI) are shown in squared brackets ([ ]). Weighting of each study is by percentage (%). p < 0.05 is deemed significant. Furthermore, for every study, the following are displayed: study author with publication date (“Study”), total sample size number for each study (“Total”), and number of clinically improved patients (“Improved”).

**Supplementary Figure 12: Complications excluding outliers**

(A-C) Forest plots visualising the pooled proportional complication rate following CSF diversion in iNPH for each surgical procedure in the order of: “Ventriculoperitoneal”, “Lumboperitoneal” and “Third-Ventriculostomy.” Meta-analysis was not performed for “Third-Ventriculostomy” due to low study number (n<3). Excluded studies identified by the influence analysis as outliers were: Grasso et al. 2023 and Hung et al. 2017, Nakajima et al. 2018 and Gangemi et al. 2008 and Pinto et al. 2013. The size of the grey square of the “Proportions” visual correlates to study sample size and the horizontal line indicates the confidence interval. The diamond at the bottom indicates the overall pooled proportion. The red bar below it indicates the prediction interval. Heterogeneity is indicated by the chi-squared statistic (I^2^) with an associated r^2^ and p-value. p-value < 0.05 is deemed significant. The 95% confidence intervals (CI) are shown in squared bracket ([ ]). Weighting of each study is by percentage (%). P < 0.05 is deemed significant. Furthermore, for every study the following are displayed: study author with publication date (“Study”), total sample size number for each study (“Total”), and number of complications (“Complications”).
